# From non-preemptive to preemptive scheduling using synchronization synthesis

**DOI:** 10.1007/s10703-016-0256-5

**Published:** 2016-09-27

**Authors:** Pavol Černý, Edmund M. Clarke, Thomas A. Henzinger, Arjun Radhakrishna, Leonid Ryzhyk, Roopsha Samanta, Thorsten Tarrach

**Affiliations:** 1 University of Colorado Boulder, 425 UCB, Boulder, CO 80309 USA; 2 Carnegie Mellon University, 5000 Forbes Avenue, Pittsburgh, PA 15213 USA; 3 IST Austria, Am Campus 1, 3400 Klosterneuburg, Austria; 4 University of Pennsylvania, 3330 Walnut Street, Philadelphia, PA 19104 USA; 5 Samsung Research America, 665 Clyde Avenue, Mountain View, CA 94043 USA; 6 University of Purdue, 610 Purdue Mall, West Lafayette, IN 47907 USA

**Keywords:** Synthesis, Concurrency, NFA language inclusion, MaxSAT

## Abstract

We present a computer-aided programming approach to concurrency. The approach allows programmers to program assuming a friendly, non-preemptive scheduler, and our synthesis procedure inserts synchronization to ensure that the final program works even with a preemptive scheduler. The correctness specification is implicit, inferred from the non-preemptive behavior. Let us consider sequences of calls that the program makes to an external interface. The specification requires that any such sequence produced under a preemptive scheduler should be included in the set of sequences produced under a non-preemptive scheduler. We guarantee that our synthesis does not introduce deadlocks and that the synchronization inserted is optimal w.r.t. a given objective function. The solution is based on a finitary abstraction, an algorithm for bounded language inclusion modulo an independence relation, and generation of a set of global constraints over synchronization placements. Each model of the global constraints set corresponds to a correctness-ensuring synchronization placement. The placement that is optimal w.r.t. the given objective function is chosen as the synchronization solution. We apply the approach to device-driver programming, where the driver threads call the software interface of the device and the API provided by the operating system. Our experiments demonstrate that our synthesis method is precise and efficient. The implicit specification helped us find one concurrency bug previously missed when model-checking using an explicit, user-provided specification. We implemented objective functions for coarse-grained and fine-grained locking and observed that different synchronization placements are produced for our experiments, favoring a minimal number of synchronization operations or maximum concurrency, respectively.

## Introduction

Programming for a concurrent shared-memory system, such as most common computing devices today, is notoriously difficult and error-prone. Program synthesis for concurrency aims to mitigate this complexity by synthesizing synchronization code automatically [5, 6, 9, 15]. However, specifying the programmer’s intent may be a challenge in itself. Declarative mechanisms, such as assertions, suffer from the drawback that it is difficult to ensure that the specification is complete and fully captures the programmer’s intent.

We propose a solution where the specification is *implicit*. We observe that a core difficulty in concurrent programming originates from the fact that the scheduler can *preempt* the execution of a thread at any time. We therefore give the developer the option to program assuming a friendly, *non-preemptive*, scheduler. Our tool automatically synthesizes synchronization code to ensure that every behavior of the program under preemptive scheduling is included in the set of behaviors produced under non-preemptive scheduling. Thus, we use the non-preemptive semantics as an implicit correctness specification.

The non-preemptive scheduling model (also known as *cooperative scheduling* [26]) can simplify the development of concurrent software, including operating system (OS) kernels, network servers, database systems, etc. [21, 22]. In the non-preemptive model, a thread can only be descheduled by voluntarily yielding control, e.g., by invoking a blocking operation. Synchronization primitives may be used for communication between threads, e.g., a producer thread may use a semaphore to notify the consumer about availability of data. However, one does not need to worry about protecting accesses to shared state: a series of memory accesses executes atomically as long as the scheduled thread does not yield.

A user evaluation by Sadowski and Yi [22] demonstrated that this model makes it easier for programmers to reason about and identify defects in concurrent code. There exist alternative implicit correctness specifications for concurrent programs. For example, for functional programs one can specify the final output of the sequential execution as the correct output. The synthesizer must then generate a concurrent program that is guaranteed to produce the same output as the sequential version [3]. This approach does not allow any form of thread coordination, e.g., threads cannot be arranged in a producer–consumer fashion. In addition, it is not applicable to reactive systems, such as device drivers, where threads are not required to terminate.

Another implicit specification technique is based on placing *atomic sections* in the source code of the program [14]. In the synthesized program the computation performed by an atomic section must appear atomic with respect to the rest of the program. Specifications based on atomic sections and specifications based on the non-preemptive scheduling model, used by our tool, can be easily expressed in terms of each other. For example, one can simulate atomic sections by placing $\mathsf {yield}$ statements before and after each atomic section, as well as around every instruction that does not belong to any atomic section.

We believe that, at least for systems code, specifications based on the non-preemptive scheduling model are easier to write and are less error-prone than atomic sections. Atomic sections are subject to syntactic constraints. Each section is marked by a pair of matching opening and closing statements, which in practice means that the section must start and end within the same program block. In contrast, a $\mathsf {yield}$ can be placed anywhere in the program.

Moreover, atomic sections restrict the use of thread synchronization primitives such as semaphores. An atomic section either executes in its entirety or not at all. In the former case, all wait conditions along the execution path through the atomic section must be simultaneously satisfied *before* the atomic section starts executing. In practice, to avoid deadlocks, one can only place a blocking instruction at the start of an atomic section. Combined with syntactic constraints discussed above, this restricts the use of thread coordination with atomic sections—a severe limitation for systems code where thread coordination is common. In contrast, synchronization primitives can be used freely under non-preemptive scheduling. Internally, they are modeled using $\mathsf {yield}$s: for instance, a semaphore acquisition instruction is modeled by a $\mathsf {yield}$ followed by an $\mathsf {assume}$ statement that proceeds when the semaphore becomes available.

Lastly, our specification defaults to the safe choice of assuming everything needs to be atomic unless a $\mathsf {yield}$ statement is placed by the programmer. In contrast, code that uses atomic sections can be preempted at any point unless protected by an explicit atomic section.

In defining behavioral equivalence between preemptive and non-preemptive executions, we focus on externally observable program behaviors: two program executions are *observationally equivalent* if they generate the same sequences of calls to interfaces of interest. This approach facilitates modular synthesis where a module’s behavior is characterized in terms of its interaction with other modules. Given a multi-threaded program $\mathscr {C}$ and a synthesized program $\mathscr {C}'$ obtained by adding synchronization to $\mathscr {C}, \mathscr {C}'$ is *preemption-safe* w.r.t. $\mathscr {C}$ if for each execution of $\mathscr {C}'$ under a preemptive scheduler, there is an observationally equivalent non-preemptive execution of $\mathscr {C}$. Our synthesis goal is to automatically generate a preemption-safe version of the input program.

We rely on abstraction to achieve efficient synthesis of multi-threaded programs. We propose a simple, *data-oblivious* abstraction inspired by an analysis of synchronization patterns in OS code, which tend to be independent of data values. The abstraction tracks types of accesses (read or write) to each memory location while ignoring their values. In addition, the abstraction tracks branching choices. Calls to an external interface are modeled as writes to a special memory location, with independent interfaces modeled as separate locations. To the best of our knowledge, our proposed abstraction is yet to be explored in the verification and synthesis literature. The abstract program is denoted as $\mathscr {C}_{\textit{abs}}$.

Two abstract program executions are observationally equivalent if they are equal modulo the classical independence relation *I* on memory accesses. This means that every sequence $\omega$ of observable actions is equivalent to a set of sequences of observable actions that are derived from $\omega$ by repeatedly commuting independent actions. Independent actions are accesses to different locations, and accesses to the same location iff they are both read accesses. Using this notion of equivalence, the notion of *preemption-safety* is extended to abstract programs.

Under abstraction, we model each thread as a nondeterministic finite automaton (NFA) over a finite alphabet, with each symbol corresponding to a read or a write to a particular variable. This enables us to construct NFAs $\mathsf {NP}_{\textit{abs}}$, representing the abstraction of the original program $\mathscr {C}$ under non-preemptive scheduling, and ${\mathsf {P}}_{abs}$, representing the abstraction of the synthesized program $\mathscr {C}'$ under preemptive scheduling. We show that preemption-safety of $\mathscr {C}'$ w.r.t. $\mathscr {C}$ is implied by preemption-safety of the abstract synthesized program $\mathscr {C}_{\textit{abs}}'$ w.r.t. the abstract original program $\mathscr {C}_{\textit{abs}}$, which, in turn, is implied by language inclusion modulo *I* of NFAs ${\mathsf {P}}_{abs}$ and $\mathsf {NP}_{\textit{abs}}$. While the problem of language inclusion modulo an independence relation is undecidable [2], we show that the antichain-based algorithm for standard language inclusion [11] can be adapted to decide a bounded version of language inclusion modulo an independence relation.

Our synthesis works in a counterexample-guided inductive synthesis (CEGIS) loop that accumulates a set of global constraints. The loop starts with a counterexample obtained from the language inclusion check. A counterexample is a sequence of locations in $\mathscr {C}_{\textit{abs}}$ such that their execution produce an observation sequence that is valid under the preemptive semantics, but not under the non-preemptive semantics. From the counterexample we infer *mutual exclusion (mutex) constraints*, which when enforced in the language inclusion check avoid returning the same counterexample again. We accumulate the mutex constraints from all counterexamples iteratively generated by the language inclusion check. Once the language inclusion check succeeds, we construct a set of global constraints using the accumulated mutex constraints and constraints for enforcing deadlock-freedom. This approach is the key difference to our previous work [4], where a greedy approach is employed that immediately places a lock to eliminate a bug. The greedy approach may result in a suboptimal lock placement with unnecessarily overlapping or nested locks.

The global approach allows us to use an objective function *f* to find an optimal lock placement w.r.t. *f* once all mutex constraints have been identified. Examples of objective functions include minimizing the number of $\mathsf {lock}$ statements (leading to coarse-grained locking) and maximizing concurrency (leading to fine-grained locking). We encode such an objective function, together with the global constraints, into a weighted maximum satisfiability (MaxSAT) problem, which is then solved using an off-the-shelf solver.

Since the synthesized lock placement is guaranteed not to introduce deadlocks our solution follows good programming practices with respect to locks: no double locking, no double unlocking and no locks locked at the end of the execution.

We implemented our synthesis procedure in a new prototype tool called Liss (Language Inclusion-based Synchronization Synthesis) and evaluated it on a series of device driver benchmarks, including an Ethernet driver for Linux and the synchronization skeleton of a USB-to-serial controller driver, as well as an in-memory key-value store server. First, Liss was able to detect and eliminate all but two known concurrency bugs in our examples; these included one bug that we previously missed when synthesizing from explicit specifications [6], due to a missing assertion. Second, our abstraction proved highly efficient: Liss runs an order of magnitude faster on the more complicated examples than our previous synthesis tool based on the CBMC model checker. Third, our coarse abstraction proved surprisingly precise for systems code: across all our benchmarks, we only encountered three program locations where manual abstraction refinement was needed to avoid the generation of unnecessary synchronization. Fourth, our tool finds a deadlock-free lock placement for both a fine-grained and a coarse-grained objective function. Overall, our evaluation strongly supports the use of the implicit specification approach based on non-preemptive scheduling semantics as well as the use of the data-oblivious abstraction to achieve practical synthesis for real-world systems code. With the two objective functions we implemented, Liss produces an optimal lock placements w.r.t. the objective.

**Contributions** First, we propose a new specification-free approach to synchronization synthesis. Given a program written assuming a friendly, non-preemptive scheduler, we automatically generate a preemption-safe version of the program without introducing deadlocks. Second, we introduce a novel abstraction scheme and use it to reduce preemption-safety to language inclusion modulo an independence relation. Third, we present the first language inclusion-based synchronization synthesis procedure and tool for concurrent programs. Our synthesis procedure includes a new algorithm for a bounded version of our inherently undecidable language inclusion problem. Fourth, we synthesize an optimal lock placement w.r.t. an objective function. Finally, we evaluate our synthesis procedure on several examples. To the best of our knowledge, Liss is the first synthesis tool capable of handling realistic (albeit simplified) device driver code, while previous tools were evaluated on small fragments of driver code or on manually extracted synchronization skeletons.

## Related work

This work is an extension of our work that appeared in CAV 2015 [4]. We included a proof for Theorem 3 that shows that language inclusion is undecidable for our particular construction of automata and independence relation. Further, we introduced a set of global mutex constraints that replace the greedy approach of our previous work and enables optimal lock placement according to an objective function.

Synthesis of synchronization is an active research area [3, 5, 6, 8, 12, 15, 17, 23, 24]. Closest to our work is a recent paper by Bloem et al. [3], which uses implicit specifications for synchronization synthesis. While their specification is given by sequential behaviors, ours is given by non-preemptive behaviors. This makes our approach applicable to scenarios where threads need to communicate explicitly. Further, correctness in Bloem et al. [3] is determined by comparing values at the end of the execution. In contrast, we compare sequences of events, which serves as a more suitable specification for infinitely-looping reactive systems. Further, Khoshnood et al. developed ConcBugAssist [18], similar to our earlier paper [15], that employs a greedy loop to fix assertion violations in concurrent programs.

Our previous work [5, 6, 15] develops the trace-based synthesis algorithm. The input is a program with assertions in the code, which represent an explicit correctness specification. The algorithm proceeds in a loop where in each iteration a faulty trace is obtained using an external model checker. A trace is faulty if it violates the specification. The trace is subsequently generalized to a partial order [5, 6] or a formula over happens-before relations [15], both representing a set of faulty traces. A formula over happens-before relations is basically a disjunction of partial orders. In our earlier previous work [5, 6] the partial order is used to synthesize atomic sections and inner-thread reorderings of independent statements. In our later work [15] the happens-before formula is used to obtain locks, wait-signal statements, and barriers. The quality of the synthesized code heavily depends on how well the generalization steps works. Intuitively the more faulty traces are removed in one synthesis step the more general the solution is and the closer it is to the solution a human would have implemented.

The drawback of assertions as a specification is that it is hard to determine if a given set of assertions represents a complete specification. The current work does not rely on an external model-checker or an explicit specification. Here we are solving language inclusion, a computationally harder problem than reachability. However, due to our abstraction, our tool performs significantly better than tools from our previous work [5, 6], which are based on a mature model checker (CBMC [10]). Our abstraction is reminiscent of previously used abstractions that track reads and writes to individual locations (e.g., [1, 25]). However, our abstraction is novel as it additionally tracks some control-flow information (specifically, the branches taken) giving us higher precision with almost negligible computational cost. For the trace generalization and synthesis we use the technique from our previous work [15] to infer looks. Due to our choice of specification no other synchronization primitives are needed.

In Vechev et al. [24] the authors rely on assertions for synchronization synthesis and include iterative abstraction refinement in their framework. This is an interesting extension to pursue for our abstraction. In other related work, CFix [17] can detect and fix concurrency bugs by identifying simple bug patterns in the code.

The concepts of linearizability and serializability are very similar to our implicit specification. Linearizability [16] describes the illusion that every method of an object takes effect instantaneously at some point between the method call and return. A set of transactions is serializable [13, 20] if they produce the same result, whether scheduled in parallel or in sequential order.

There has been a body of work on using a non-preemptive (cooperative) scheduler as an implicit specification. The notion of cooperability was introduced by Yi and Flanagan [26]. They require the user to annotate the program with yield statements to indicate thread interference. Then their system verifies that the yield specification is complete meaning that every trace is cooperable. A preemptive trace is cooperable if it is equivalent to a trace under the cooperative scheduler.

**Fig. 1:**
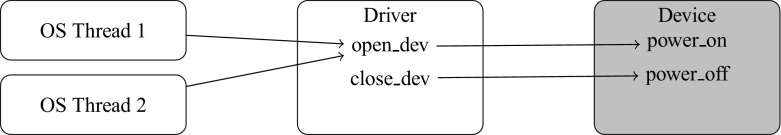
Interaction of the device driver with the OS and the device

**Fig. 2:**
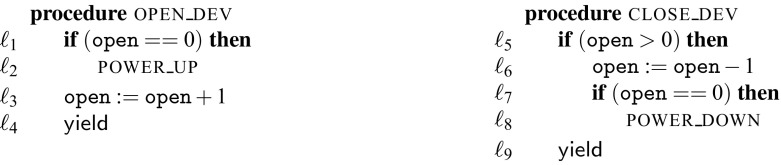
Running example

**Fig. 3:**
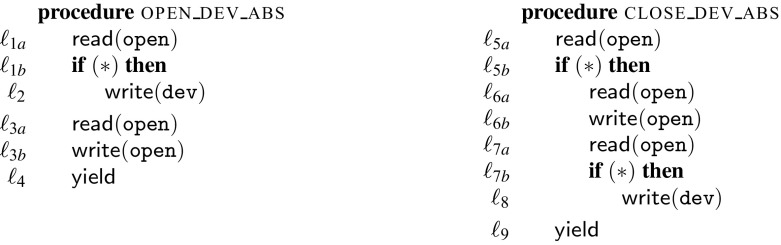
Abstraction of the running example

## Illustrative example

Figure 2 contains our running example, a part of a device driver. A driver interfaces the operating system with the hardware device (as illustrated in Fig. 1) and may be used by different threads of the operating system in parallel. An operating system thread wishing to use the device must first call the open_dev procedure and finally the close_dev procedure to indicate it no longer needs the device. The driver keeps track of the number of threads that interact with the device. The first thread to call open_dev will cause the driver to power up the device, the last thread to call close_dev will cause the driver to power down the device. The interaction between the driver and the device are represented as procedure calls in lines $\ell _2$ and $\ell _8$. From the device’s perspective, the power-on and power-off signals alternate. In general, we must assume that it is not safe to send the power-on signal twice in a row to the device. If executed with the non-preemptive scheduler the code in Fig. 2 will produce a sequence of a power-on signal followed by a power-off signal followed by a power-on signal and so on.

Consider the case where the procedure open_dev is called in parallel by two operating system threads that want to initiate usage of the device. Without additional synchronization, there could be two calls to power_up in a row when executing under a preemptive scheduler. Consider two threads ($\mathtt {T}1$ and $\mathtt {T}2$) running the open_dev procedure. The corresponding trace is $\mathtt {T}1.\ell _1;\ \mathtt {T}2.\ell _1;\ \mathtt {T}1.\ell _2;\ \mathtt {T}2.\ell _2;\ \mathtt {T}2.\ell _3;\ \mathtt {T}2.\ell _4;\ \mathtt {T}1.\ell _3;\ \mathtt {T}1.\ell _4$. This sequence is not observationally equivalent to any sequence that can be produced when executing with a non-preemptive scheduler.

Figure 3 contains the abstracted versions of the two procedures, open_dev_abs and close_dev_abs. For instance, the instruction $\mathtt {open} := \mathtt {open} + 1$ is abstracted to the two instructions labeled $\ell _{3a}$ and $\ell _{3b}$. The calls to the device (power_up and power_down) are abstracted as writes to a hypothetical $\mathtt {dev}$ variable. This expresses the fact that interactions with the device are never independent. The abstraction is coarse, but still captures the problem. Consider two threads ($\mathtt {T}1$ and $\mathtt {T}2$) running the open_dev_abs procedure. The following trace is possible under a preemptive scheduler, but not under a non-preemptive scheduler: $\mathtt {T}1.\ell _{1a};\ \mathtt {T}1.\ell _{1b};\ \mathtt {T}2.\ell _{1a};\ \mathtt {T}2.\ell _{1b};\ \mathtt {T}1.\ell _2;\ \mathtt {T}2.\ell _2;\ \mathtt {T}2.\ell _{3a};\ \mathtt {T}2.\ell _{3b};\ \mathtt {T}2.\ell _4;\ \mathtt {T}1.\ell _{3a};\ \mathtt {T}1.\ell _{3b};\ \mathtt {T}1.\ell _4$. Moreover, the trace cannot be transformed by swapping independent events into any trace possible under a non-preemptive scheduler. This is because instructions $\ell _{3b}:\mathsf {write}(\mathtt {open})$ and $\ell _{1a}:\mathsf {read}(\mathtt {open})$ are not independent. Further, $\ell _2:\mathsf {write}(\mathtt {dev})$ is not independent with itself. Hence, the abstract trace exhibits the problem of two successive calls to power_up when executing with a preemptive scheduler. Our synthesis procedure finds this problem, and stores it as a mutex constraint: $\mathrm {mtx}([\ell _{1a}{:}\ell _{3b}],[\ell _2{:}\ell _{3b}])$. Intuitively this constraint expresses the fact if one thread is executing any instruction between $\ell _{1a}$ and $\ell _{3b}$ no other thread may execute $\ell _2$ or $\ell _{3b}$.

**Fig. 4:**
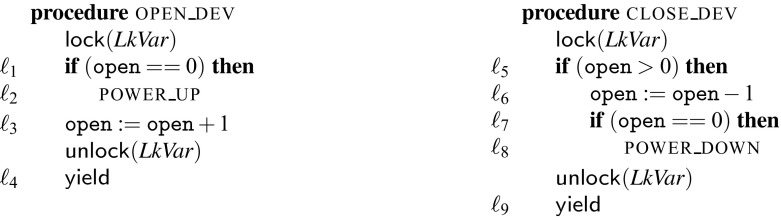
Running example with the synthesized locks

While this constraint ensures two parallel calls to open_dev behave correctly, two parallel calls to close_dev may result in the device receiving two power_down signals. This is represented by the concrete trace $\mathtt {T}1.\ell _5;\ \mathtt {T}1.\ell _6;\ \mathtt {T}2.\ell _5;\ \mathtt {T}2.\ell _6;\ \mathtt {T}2.\ell _7;\ \mathtt {T}2.\ell _8;\ \mathtt {T}2.\ell _9;\ \mathtt {T}1.\ell _7;\ \mathtt {T}1.\ell _8;\ \mathtt {T}1.\ell _9$. The corresponding abstract trace is $\mathtt {T}1.\ell _{5a};\ \mathtt {T}1.\ell _{5b};\ \mathtt {T}1.\ell _{6a};\ \mathtt {T}1.\ell _{6b};\ \mathtt {T}2.\ell _{5a};\ \mathtt {T}2.\ell _{5b};\ \mathtt {T}2.\ell _{6a};\ \mathtt {T}2.\ell _{6b};\ \mathtt {T}2.\ell _{7a};\ \mathtt {T}2.\ell _{7b};\ \mathtt {T}2.\ell _8;\ \mathtt {T}2.\ell _9;\ \mathtt {T}1.\ell _{7a};\ \mathtt {T}1.\ell _{7b};\ \mathtt {T}1.\ell _8;\ \mathtt {T}1.\ell _9$. This trace is not possible under a non-preemptive scheduler and cannot be transformed to a trace possible under a non-preemptive scheduler. This results in a second mutex constraint $\mathrm {mtx}([\ell _{5a}{:}\ell _{8}],[\ell _{6b}{:}\ell _{8}])$. With both mutex constraints the program is correct. Our lock placement procedure then encodes these constraints in SMT and the models of the SMT formula are all the correct lock placements. In Fig. 4 we show open_dev and close_dev with the inserted locks.

## Formal framework and problem statement

We present the syntax and semantics of a *concrete* concurrent while language ${\mathscr {W}}$. For our solution strategy to be efficient we require an abstraction and we also introduce the syntax and semantics of the *abstract* concurrent while language ${{\mathscr {W}}}_{\textit{abs}}$. While ${\mathscr {W}}$ (and our tool) permits non-recursive function call and return statements, we skip these constructs in the formalization below. We conclude the section by formalizing our notion of correctness for concrete concurrent programs.

### Concrete concurrent programs

In our work, we assume a read or a write to a single shared variable executes atomically and further assume a sequentially consistent memory model.

#### Syntax of ${\mathscr {W}}$ (Fig. 5)

A concurrent program is a finite collection of threads $\langle \mathtt {T}1, \ldots , \mathtt {T}n \rangle$ where each thread is a statement written in the syntax of ${\mathscr {W}}$. Variables in ${\mathscr {W}}$ can be categorized into

- shared variables $$ ShVar _i$$,
- thread-local variables $$ LoVar _i$$,
- lock variables $$ LkVar _i$$,
- condition variables $$ CondVar _i$$ for wait-signal statements, and
- guard variables $$ GrdVar _i$$ for assumptions.

The $$ LkVar _i, CondVar _i$$ and $$ GrdVar _i$$ variables are also shared between all threads. All variables range over integers with the exception of guard variables that range over Booleans ($$\mathtt {true},\mathtt {false}$$). Each statement is labeled with a unique location identifier $$ \ell $$; we denote by $$\mathsf {stmt}({\ell })$$ the statement labeled by $$\ell $$.

The language ${\mathscr {W}}$ includes standard sequential constructs, such as assignments, loops, conditionals, and $\mathsf {goto}$ statements. Additional statements control the interaction between threads, such as lock, wait-notify, and $\mathsf {yield}$ statements. In ${\mathscr {W}}$, we only permit expressions that read from at most one shared variable and assignments that either read from or write to exactly one shared variable.^1^ The language also includes $\mathsf {assume}$, $\mathsf {assume\_not}$ statements that operate on guard variables and become relevant later for our abstraction. The $\mathsf {yield}$ statement is in a sense an annotation as it has no effect on the actual program running under a preemptive scheduler. We still present it here because it has a semantic meaning under the non-preemptive scheduler.

Language ${\mathscr {W}}$ has two statements that allow communication with an external system: $\mathsf {input}( ch )$ reads from and $\mathsf {output}( ch , ShExp )$ writes to a communication channel $ch$. The channel is an interface between the program and an external system. The external system cannot observe the internal state of the program and only observes the information flow on the channel. In practice, we use the channels to model device registers. A device register is a special memory address, reading and writing from and to it is visible to the device. This is used to exchange information with a device. In our presentation, we assume all channels communicate with the same external system.

**Fig. 5:**
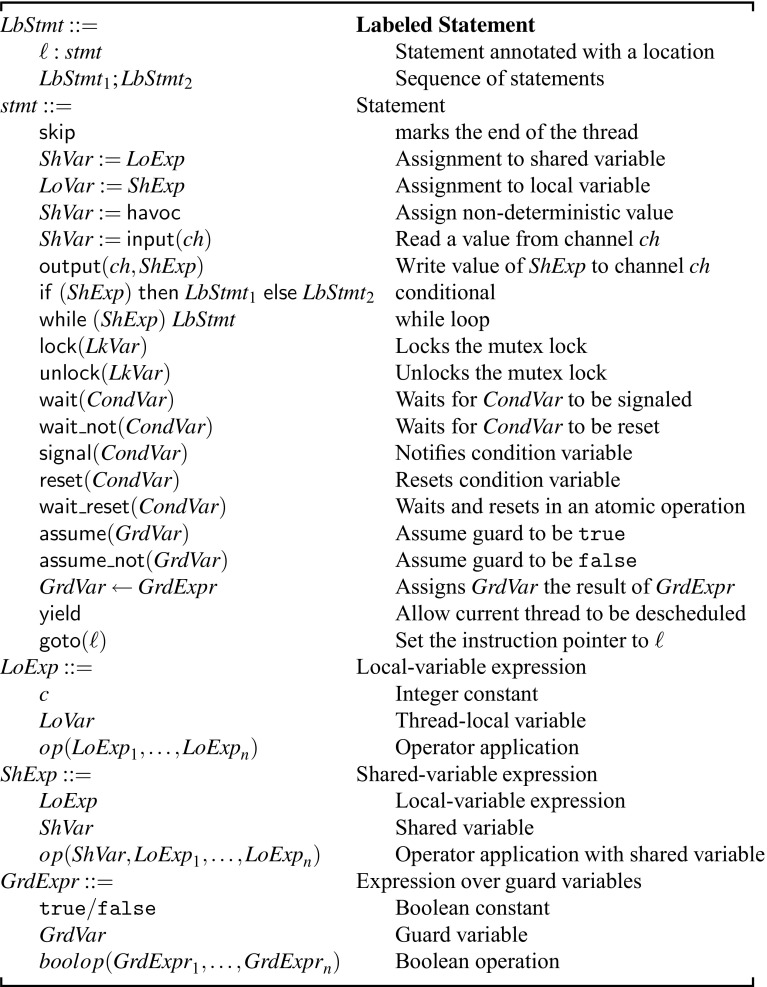
Syntax of $${\mathscr {W}}$$

#### Semantics of ${\mathscr {W}}$

We first define the semantics of a single thread in ${\mathscr {W}}$, and then extend the definition to concurrent non-preemptive and preemptive semantics.

##### 4.1.2.1 Single-thread semantics (Fig. 6)

Let us fix a thread identifier $tid$. We use $tid$ interchangeably with the program it represents. A state of a single thread is given by $\langle \mathscr {V}, \ell \rangle$ where $\mathscr {V}$ is a valuation of all program variables, and $\ell$ is a location identifier, indicating the statement in $tid$ to be executed next. A thread is guaranteed not to read or write thread-local variables of other threads.

We define the *flow graph* ${\mathscr {G}}_{ tid }$ for thread $tid$ in a manner similar to the control-flow graph of $tid$. Every node of ${\mathscr {G}}_{ tid }$ represents a single statement (basic blocks are not merged) and the node is labeled with the location $\ell$ of the statement. The flow graph ${\mathscr {G}}_{ tid }$ has a unique entry node and a unique exit node. These two may coincide if the thread has no statements. The entry node is the first labeled statement in $tid$; we denote its location identifier by $\mathsf{first}_{ tid }$. The exit node is a special node corresponding to a hypothetical statement $\mathsf{last}_{ tid }:\, \mathsf {skip}$ placed at the end of $tid$.

We define successors of locations of $tid$ using ${\mathscr {G}}_{ tid }$. The location last has no successors. We define $\mathsf{succ}(\ell ) = \ell '$ if node $\ell : stmt$ in ${\mathscr {G}}_{ tid }$ has exactly one outgoing edge to node $\ell ': stmt '$. Nodes representing conditionals and loops have two outgoing edges. We define $\mathsf{succ}_1(\ell ) = \ell _1$ and $\mathsf{succ}_2(\ell ) = \ell _2$ if node $\ell : stmt$ in ${\mathscr {G}}_{ tid }$ has exactly two outgoing edges to nodes $\ell _1: stmt _1$ and $\ell _2: stmt _2$. Here $\mathsf{succ}_1$ represents the $\mathsf {then}$ or the $\mathsf {loop}$ branch, whereas $\mathsf{succ}_2$ represents the $\mathsf {else}$ or the $\mathsf {loopexit}$ branch.

We can now define the single-thread operational semantics. A single execution step $\langle \mathscr {V}, \ell \rangle \xrightarrow {\alpha } \langle \mathscr {V}', \ell ' \rangle$ changes the program state from $\langle \mathscr {V}, \ell \rangle$ to $\langle \mathscr {V}', \ell '\rangle$, while optionally outputting an *observable symbol* $\alpha$. The absence of a symbol is denoted using $\epsilon$. In the following, $e$ represents an expression and $e[v / \mathscr {V}[v]]$ evaluates an expression by replacing all variables *v* with their values in $\mathscr {V}$. We use $\mathscr {V}[v:=k]$ to denote that variable *v* is set to *k* and all other variables in $\mathscr {V}$ remain unchanged.

**Fig. 6:**
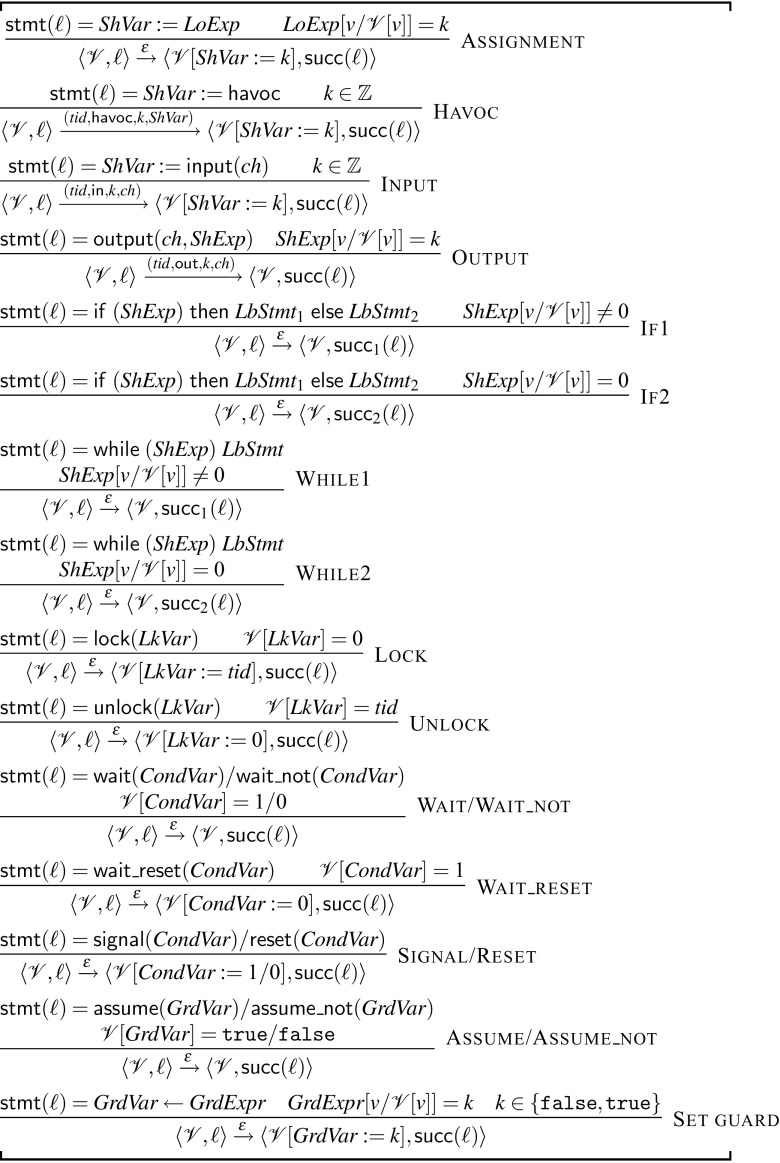
Single-thread semantics of ${\mathscr {W}}$

In Fig. 6, we present the rules for single execution steps. Each step is atomic, no interference can occur while the expressions in the premise are being evaluated. The only rules with an observable output are:

1. Havoc: Statement $\ell : ShVar :=\mathsf {havoc}$ assigns shared variable $ShVar$ a non-deterministic value (say *k*) and outputs the observable $( tid , \mathsf {havoc}, k, ShVar )$.
2. Input, Output: $\ell : ShVar :=\mathsf {input}( ch )$ and $\ell : \mathsf {output}( ch , ShExp )$ read and write values to the channel $ch$, and output $( tid , \mathsf {in}, k, ch )$ and $( tid , \mathsf {out}, k, ch )$, where *k* is the value read or written, respectively.

Intuitively, the observables record the sequence of non-deterministic guesses, as well as the input/output interaction with the tagged channels. The semantics of the synchronization statements shown in Fig. 6 is standard. The lock and unlock statements do not count and do not allow double (un)locking. There are no rules for $\mathsf {goto}$ and the sequence statement because they are already taken care of by the flow graph.

#### Concurrent semantics

A state of a concurrent program is given by $\langle \mathscr {V}, \textit{ctid}, (\ell _1, \ldots , \ell _n) \rangle$ where $\mathscr {V}$ is a valuation of all program variables, $\textit{ctid}$ is the thread identifier of the currently executing thread and $\ell _1, \ldots , \ell _n$ are the locations of the statements to be executed next in threads $\mathtt {T}_1$ to $\mathtt {T}_n$, respectively. There are two additional states: $\langle \mathtt {terminated}\rangle$ indicates the program has finished and $\langle \mathtt {failed}\rangle$ indicates an assumption failed. Initially, all integer program variables and $\textit{ctid}$ equal 0, all guard variable equal $\mathtt {false}$ and for each $i \in [1,n]: \ell _i = \mathtt {first}_i$. We introduce a non-preemptive and a preemptive semantics. The former is used as a specification of allowed executions, whereas the latter models concurrent sequentially consistent executions of the program.

**Fig. 7:**
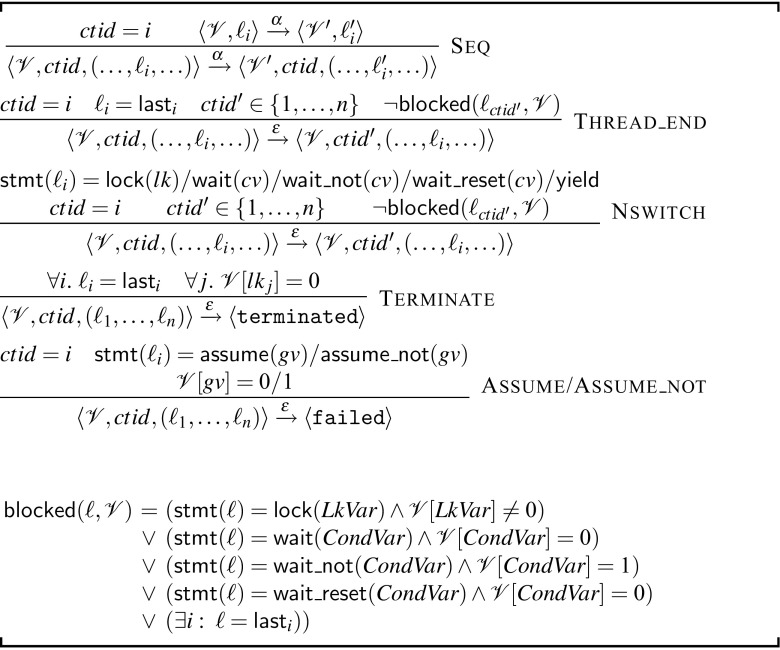
Non-preemptive semantics

*4.1.3.1 Non-preemptive semantics (Fig.* 7 *)* The non-preemptive semantics ensures that a single thread from the program keeps executing using the single-thread semantics (Rule Seq) until one of the following occurs: (a) the thread finishes execution (Rule Thread_end) or (b) it encounters a $\mathsf {yield}$, $\mathsf {lock}$, $\mathsf {wait}$ or $\mathsf {wait\_not}$ statement (Rule Nswitch). In these cases, a context-switch is possible, however, the new thread must not be blocked. We consider a thread blocked if its current instruction is to acquire an unavailable lock, waits for a condition that is not signaled, or the thread reached the $\mathsf{last}$ location. Note the difference between $\mathsf {wait}$/$\mathsf {wait\_not}$ and $\mathsf {assume}$/$\mathsf {assume\_not}$. The former allow for a context-switch while the latter transitions to the $\langle \mathtt {failed}\rangle$ state if the assume is not fulfilled (rule Assume/Assume_not). A special rule exists for termination (Rule Terminate), which requires that all threads finished execution and also all locks are unlocked.

**Fig. 8:**
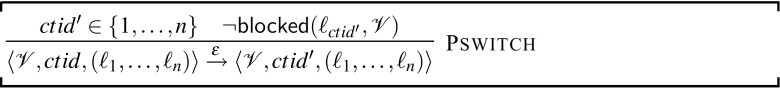
Additional rule for preemptive semantics

*4.1.3.2 Preemptive semantics (Figs.* 7, 8 *)* The preemptive semantics of a program is obtained from the non-preemptive semantics by relaxing the condition on context-switches, and allowing context-switches at all program points. In particular, the preemptive semantics consist of the rules of the non-preemptive semantics and the single rule Pswitch in Fig. 8.

### Abstract concurrent programs

The state of the concrete semantics contains unbounded integer variables, which may result in an infinite state space. We therefore introduce a simple, data-oblivious abstraction ${{\mathscr {W}}}_{\textit{abs}}$ for concurrent programs written in ${\mathscr {W}}$ communicating with an external system. The abstraction tracks types of accesses (read or write) to each memory location while abstracting away their values. Inputs/outputs to a channel are modeled as writes to a special memory location ($\mathtt{dev}$). Even inputs are modeled as writes because in our applications we cannot assume that reads from the external interface are free of side-effects in the component on the other side of the interface. Havocs become ordinary writes to the variable they are assigned to. Every branch is taken non-deterministically and tracked. Given $\mathscr {C}$ written in ${\mathscr {W}}$, we denote by $\mathscr {C}_{\textit{abs}}$ the corresponding abstract program written in ${{\mathscr {W}}}_{\textit{abs}}$.

#### Abstract syntax (Fig. 9)

In the figure, $var$ denotes all shared program variables and the $\mathtt{dev}$ variable. The syntax of all synchronization primitives and the assumptions over guard variables remains unchanged. The purpose of the guard variables is to improve the precision of our otherwise coarse abstraction. Currently, they are inferred manually, but can presumably be inferred automatically using an iterative abstraction-refinement loop. In our current benchmarks, guard variables needed to be introduced in only three scenarios.

**Fig. 9:**
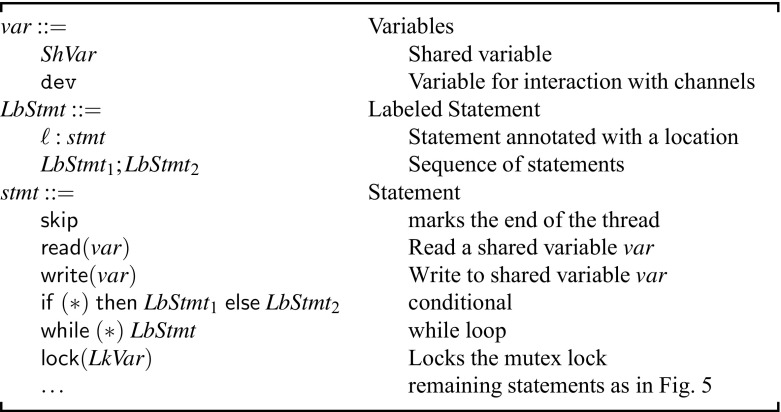
Syntax of $${{\mathscr {W}}}_{\textit{abs}}$$

#### Abstraction function (Fig. 10)

A thread in ${\mathscr {W}}$ can be translated to ${{\mathscr {W}}}_{\textit{abs}}$ using the abstraction function . The abstraction replaces all global variable access with $\mathsf {read}( var )$ and $\mathsf {write}( var )$ and replaces branching conditions with nondeterminism ($*$). All synchronization primitives remain unaffected by the abstraction. The abstraction may result in duplicate labels $\ell$, which are replaced by fresh labels. $\mathsf {goto}$ statements are reordered accordingly. Our abstraction records branching choices (branch tagging). If one were to remove branch-tagging, the abstraction would be unsound. The justification and intuition for this can be found further below in Theorem 1. For example in our running example in Fig. 2 the abstraction of $\ell _1$ results in two abstract labels $\ell _{1a}$ and $\ell _{1b}$ in Fig. 3.

**Fig. 10:**
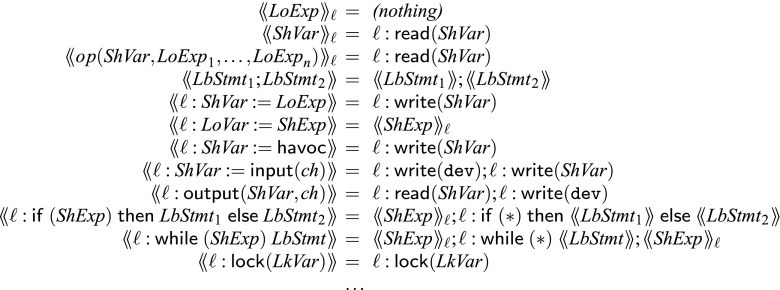
Abstraction function from ${\mathscr {W}}$ to ${{\mathscr {W}}}_{\textit{abs}}$

#### Abstract semantics

As before, we first define the semantics of ${{\mathscr {W}}}_{\textit{abs}}$ for a single-thread.

##### 4.2.3.1 Single-thread semantics (Fig. 11)

**Fig. 11:**
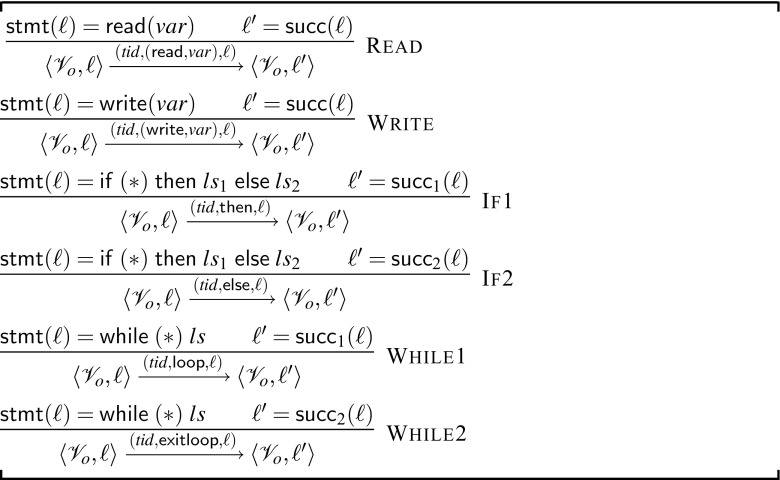
Partial set of rules for single-thread semantics of ${{\mathscr {W}}}_{\textit{abs}}$

The abstract state of a single thread $tid$ is given simply by $\langle \mathscr {V}_o,\ell \rangle$ where $\mathscr {V}_o$ is a valuation of all lock, condition and guard variables and $\ell$ is the location of the statement in $tid$ to be executed next. We define the flow graph and successors for locations in the abstract program $tid$ in the same way as before. An abstract observable symbol is of the form: $( tid , \theta ,\ell )$, where $\theta \in \{(\mathsf {read}, ShVar ),(\mathsf {write}, ShVar ),\mathsf {then},\mathsf {else},\mathsf {loop},\mathsf {exitloop}\}$. The symbol $\theta$ records the type of access to variables along with the variable name $((\mathsf {read}, v),(\mathsf {write}, v))$ and records non-deterministic branching choices $\{\mathsf {if},\mathsf {else},\mathsf {loop},\mathsf {exitloop}\}$. Fig. 11 presents the rules for statements unique to ${{\mathscr {W}}}_{\textit{abs}}$; the rules for statements common to ${{\mathscr {W}}}_{\textit{abs}}$ and ${\mathscr {W}}$ are the same.

##### 4.2.3.2 Concurrent semantics

A state of an abstract concurrent program is either $\langle \mathtt {terminated}\rangle , \langle \mathtt {failed}\rangle$, or is given by $\langle \mathscr {V}_o, \textit{ctid}, (\ell _1, \ldots , \ell _n) \rangle$ where $\mathscr {V}_o$ is a valuation of all lock, condition and guard variables, $\textit{ctid}$ is the current thread identifier and $\ell _1, \ldots , \ell _n$ are the locations of the statements to be executed next in threads $\mathtt {T}_1$ to $\mathtt {T}_n$, respectively. The non-preemptive and preemptive semantics of a concurrent program written in ${{\mathscr {W}}}_{\textit{abs}}$ are defined in the same way as that of a concurrent program written in ${\mathscr {W}}$.

### Program correctness and problem statement

Let $\mathbb {W}, \mathbb {W}_{\textit{abs}}$ denote the set of all concurrent programs in ${\mathscr {W}}, {{\mathscr {W}}}_{\textit{abs}}$, respectively.

#### Executions

A *non-preemptive/preemptive execution* of a concurrent program $\mathscr {C}$ in $\mathbb {W}$ is an alternating sequence of program states and (possibly empty) observable symbols, $S_0 \alpha _1 S_1 \ldots \alpha _k S_k$, such that (a) $S_0$ is the initial state of $\mathscr {C}$, (b) $\forall j \in [0,k-1]$, according to the non-preemptive/preemptive semantics of ${\mathscr {W}}$, we have $S_j \xrightarrow {\alpha _{j+1}}{S_{j+1}}$, and (c) $S_k$ is the state $\langle \mathtt {terminated}\rangle$. A non-preemptive/preemptive execution of a concurrent program $\mathscr {C}_{\textit{abs}}$ in $\mathbb {W}_{\textit{abs}}$ is defined in the same way, replacing the corresponding semantics of ${\mathscr {W}}$ with that of ${{\mathscr {W}}}_{\textit{abs}}$.

#### Observable behaviors

Let $\pi$ be an execution of program $\mathscr {C}$ in $\mathbb {W}$, then we denote with $\omega = \mathsf {obs}(\pi )$ the sequence of non-empty observable symbols in $\pi$. We use $[\![ \mathscr {C} ]\!]^{\textit{NP}}$, resp. $[\![ \mathscr {C} ]\!]^{P}$, to denote the *non-preemptive*, resp. *preemptive*, *observable behavior* of $\mathscr {C}$, that is all sequences $\mathsf {obs}(\pi )$ of all executions $\pi$ under the non-preemptive, resp. preemptive, scheduling. The *non-preemptive*/*preemptive observable behavior* of program $\mathscr {C}_{\textit{abs}}$ in $\mathbb {W}_{\textit{abs}}$, denoted $[\![ \mathscr {C}_{\textit{abs}} ]\!]^{\textit{NP}}$/$[\![ \mathscr {C}_{\textit{abs}} ]\!]^{P}$, is defined similarly.

We specify correctness of concurrent programs in $\mathbb {W}$ using two *implicit* criteria, presented below.

#### Preemption-safety

Observable behaviors $\omega _1$ and $\omega _2$ of a program $\mathscr {C}$ in $\mathbb {W}$ are *equivalent* if: (a) the subsequences of $\omega _1$ and $\omega _2$ containing only symbols of the form $( tid , \mathsf {in}, k, t)$ and $( tid , \mathsf {out}, k, t)$ are equal and (b) for each thread identifier $tid$, the subsequences of $\omega _1$ and $\omega _2$ containing only symbols of the form $( tid , \mathsf {havoc}, k, x)$ are equal. Intuitively, observable behaviors are equivalent if they have the same interaction with the interface, and the same non-deterministic choices in each thread. For sets $\mathscr {O}_1$ and $\mathscr {O}_2$ of observable behaviors, we write $\mathscr {O}_1 \Subset \mathscr {O}_2$ to denote that each sequence in $\mathscr {O}_1$ has an equivalent sequence in $\mathscr {O}_2$.

Given concurrent programs $\mathscr {C}$ and $\mathscr {C}'$ in $\mathbb {W}$ such that $\mathscr {C}'$ is obtained by adding locks to $\mathscr {C}, \mathscr {C}'$ is *preemption-safe* w.r.t. $\mathscr {C}$ if $[\![ \mathscr {C}' ]\!]^{P} \Subset [\![ \mathscr {C} ]\!]^{\textit{NP}}$.

#### Deadlock-freedom

A state $S$ of concurrent program $\mathscr {C}$ in $\mathbb {W}$ is a *deadlock state* under non-preemptive/preemptive semantics if

- The repeated application of the rules of the non-preemptive/preemptive semantics from the initial state $S_0$ of $\mathscr {C}$ can lead to $S$,
- $$S \ne \langle \mathtt {terminated}\rangle $$,
- $$S \ne \langle \mathtt {failed}\rangle $$, and
- $\lnot \exists S'$: $\langle S \rangle \xrightarrow {\alpha } \langle S' \rangle$ according to the non-preemptive/preemptive semantics of ${\mathscr {W}}$.

Program $\mathscr {C}$ in $\mathbb {W}$ is *deadlock-free under non-preemptive/preemptive semantics* if no non-preemptive/preemptive execution of $\mathscr {C}$ hits a deadlock state. In other words, every non-preemptive/preemptive execution of $\mathscr {C}$ ends in state $\langle \mathtt {terminated}\rangle$ or $\langle \mathtt {failed}\rangle$. The $\langle \mathtt {failed}\rangle$ state indicates an assumption did not hold, which we do not consider a deadlock. We say $\mathscr {C}$ is *deadlock-free* if it is deadlock-free under both non-preemptive and preemptive semantics.

#### Problem statement

We are now ready to state our main problem, the optimal synchronization synthesis problem. We assume we are given a cost function *f* from a program $\mathscr {C}'$ to the cost of the lock placement solution, formally $f:\mathbb {W}\mapsto \mathbb {R}$. Then, given a concurrent program $\mathscr {C}$ in $\mathbb {W}$, the goal is to synthesize a new concurrent program $\mathscr {C}'$ in $\mathbb {W}$ such that:

- $\mathscr {C}'$ is obtained by adding locks to $\mathscr {C}$,
- $\mathscr {C}'$ is preemption-safe w.r.t. $\mathscr {C}$,
- $\mathscr {C}'$ has no deadlocks not present in $\mathscr {C}$, *and*,
- $$\mathscr {C}' = \underset{\mathscr {C}''\in \mathbb {W}\text { satisfying (a)-(c) above}}{\arg \,\min } \; f(\mathscr {C}'')$$

**Fig. 12:**
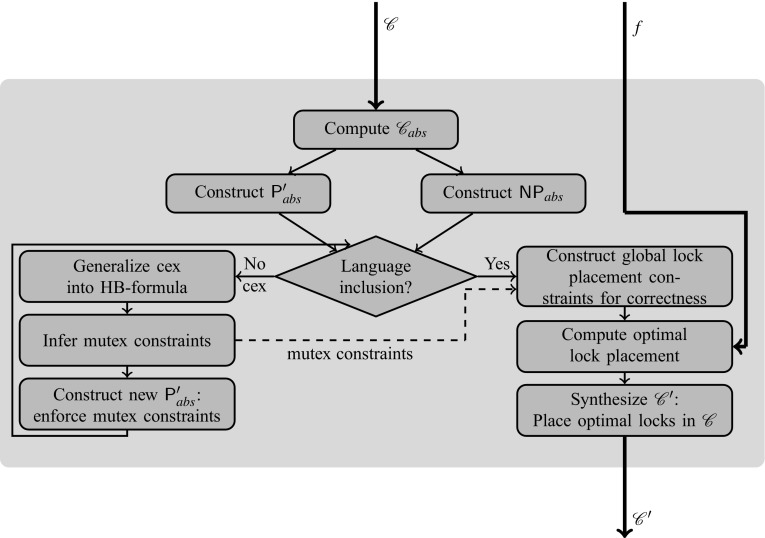
Solution overview

## Solution overview

Our solution framework (Fig. 12) consists of the following main components. We briefly describe each component below and then present them in more detail in subsequent sections.

### Reduction of preemption-safety to language inclusion

To ensure tractability of checking preemption-safety, we build the abstract program $\mathscr {C}_{\textit{abs}}$ from $\mathscr {C}$ using the abstraction function described in Sect. 4.2. Under abstraction, we model each thread as a nondeterministic finite automaton (NFA) over a finite alphabet consisting of abstract observable symbols. This enables us to construct NFAs $\mathsf {NP}_{\textit{abs}}$ and ${\mathsf {P}}'_{\textit{abs}}$ accepting the languages $[\![ \mathscr {C}_{\textit{abs}} ]\!]^{\textit{NP}}$ and $[\![ \mathscr {C}_{\textit{abs}}' ]\!]^{P}$, respectively. We proceed to check if all words of ${\mathsf {P}}'_{\textit{abs}}$ are included in $\mathsf {NP}_{\textit{abs}}$ modulo an independence relation *I* that respects the equivalence of observables. We describe the reduction of preemption-safety to language inclusion and our language inclusion check procedure in Sect. 6.

### Inference of mutex constraints from generalized counterexamples

If ${\mathsf {P}}'_{\textit{abs}}$ and $\mathsf {NP}_{\textit{abs}}$ do not satisfy language inclusion modulo *I*, then we obtain a counterexample $\textit{cex}$. A counterexample is a sequence of locations an observation sequence that is in $[\![ \mathscr {C}_{\textit{abs}} ]\!]^{P}$, but not in $[\![ \mathscr {C}_{\textit{abs}}' ]\!]^{\textit{NP}}$. We analyze $\textit{cex}$ to infer constraints on ${\mathscr {L}}({\mathsf {P}}'_{\textit{abs}})$ for eliminating $\textit{cex}$. We use $\textit{nhood}(\textit{cex})$ to denote the set of all permutations of the symbols in $\textit{cex}$ that are accepted by ${\mathsf {P}}'_{\textit{abs}}$. Our counterexample analysis examines the set $\textit{nhood}(\textit{cex})$ to obtain an *hbformula* $\phi$—a Boolean combination of *happens-before* ordering constraints between events—representing all counterexamples in $\textit{nhood}(\textit{cex})$. Thus $\textit{cex}$ is generalized into a larger set of counterexamples represented as $\phi$. From $\phi$, we infer possible *mutual exclusion (mutex) constraints* on ${\mathscr {L}}({\mathsf {P}}'_{\textit{abs}})$ that can eliminate all counterexamples satisfying $\phi$. We describe the procedure for finding constraints from $\textit{cex}$ in Sect. 7.1.

### Automaton modification for enforcing mutex constraints

Once we have the mutex constraints inferred from a generalized counterexample, we enforce them in ${\mathsf {P}}'_{\textit{abs}}$, effectively removing transitions from the automaton that violate the mutex constraint. This completes our loop and we repeat the language inclusion check of ${\mathsf {P}}'_{\textit{abs}}$ and $\mathsf {NP}_{\textit{abs}}$. If another counterexample is found our loop continues, if the language inclusion check succeeds we proceed to the lock placement. This differs from the greedy approach employed in our previous work [4] that modifies $\mathscr {C}_{\textit{abs}}'$ and then constructs a new automaton ${\mathsf {P}}'_{\textit{abs}}$ from $\mathscr {C}_{\textit{abs}}'$ before restarting the language inclusion. The greedy approach inserts locks into $\mathscr {C}_{\textit{abs}}'$ that are never removed in a future iteration. This can lead to inefficient lock placement. For example a larger lock may be placed that completely surrounds an earlier placed lock.

### Computation of an *f*-optimal lock placement

Once ${\mathsf {P}}'_{\textit{abs}}$ and $\mathsf {NP}_{\textit{abs}}$ satisfy language inclusion modulo *I*, we formulate global constraints over lock placements for ensuring correctness. These global constraints include all mutex constraints inferred over all iterations and constraints for enforcing deadlock-freedom. Any model of the global constraints corresponds to a lock placement that ensures program correctness. We describe the formulation of these global constraints in Sect. 8.

Given a cost function *f*, we compute a lock placement that satisfies the global constraints and is optimal w.r.t. *f*. We then synthesize the final output $\mathscr {C}'$ by inserting the computed lock placement in $\mathscr {C}$. We present various objective functions and describe the computation of their respective optimal solutions in Sect. 9.

## Checking preemption-safety

### Reduction of preemption-safety to language inclusion

#### Soundness of the abstraction

Formally, two observable behaviors $\omega _1=\alpha _0\ldots \alpha _k$ and $\omega _2=\beta _0\ldots \beta _k$ of an abstract program $\mathscr {C}_{\textit{abs}}$ in $\mathbb {W}_{\textit{abs}}$ are *equivalent* if:

1. For each thread $tid$, the subsequences of $\alpha _0\ldots \alpha _k$ and $\beta _0\ldots \beta _k$ containing only symbols of the form $( tid ,a,\ell )$, for all *a*, are equal,
2. For each variable *var*, the subsequences of $\alpha _0\ldots \alpha _k$ and $\beta _0\ldots \beta _k$ containing only write symbols (of the form $( tid , (\mathsf {write}, var), \ell )$) are equal, and
3. For each variable *var*, the multisets of symbols of the form $( tid , (\mathsf {read}, var), \ell )$ between any two write symbols, as well as before the first write symbol and after the last write symbol are identical.

Using this notion of equivalence, the notion of preemption-safety is extended to abstract programs: Given abstract concurrent programs $\mathscr {C}_{abs}$ and $\mathscr {C}'_{abs}$ in $\mathbb {W}_{abs}$ such that $\mathscr {C}'_{abs}$ is obtained by adding locks to $\mathscr {C}_{abs}, \mathscr {C}'_{abs}$ is *preemption-safe* w.r.t. $\mathscr {C}_{abs}$ if $[\![ \mathscr {C}'_{abs} ]\!]^{P} \Subset _{abs} [\![ \mathscr {C}_{abs} ]\!]^{\textit{NP}}$.

For the abstraction to be sound we require only that whenever preemption-safety does not hold for a program $\mathscr {C}$, then there must be a trace in its abstraction $\mathscr {C}_{\textit{abs}}$ feasible under preemptive, but not under non-preemptive semantics.

**Fig. 13:**
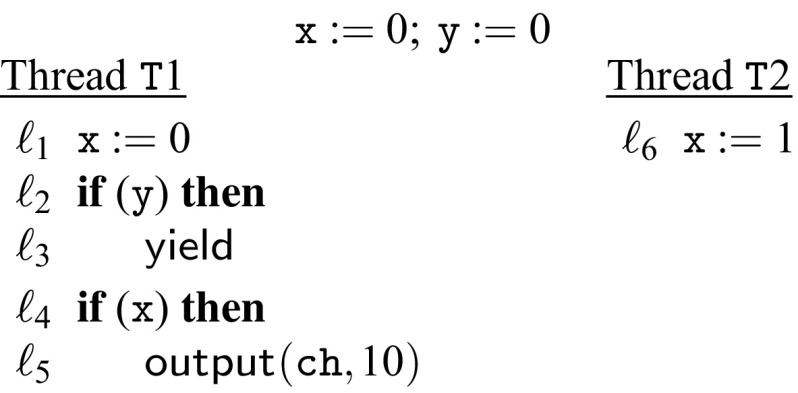
Example showing how the abstraction works

To illustrate this we use the program in Fig. 13, which is not preemption-safe. To see this consider the observation $(\mathtt {T1},\mathsf {out}, 10, \mathtt{ch})$ that cannot occur in the non-preemptive semantics because x is always 0 at $\ell _4$. Note that $\ell _3$ is unreachable because the variable y is initialized to 0 and never assigned. With the preemptive semantics the output can be observed if thread $\mathtt {T}2$ interrupts thread $\mathtt {T}1$ between lines $\ell _1$ and $\ell _4$. An example trace would be $\ell _1;\ \ell _6;\ \ell _2;\ \ell _4;\ \ell _5$.

If we consider the abstract semantics, we notice that under the non-preemptive abstract semantics $\ell _3$ is reachable because the abstraction makes the branching condition in $\ell _2$ non-deterministic. However, since our abstraction is sound there must still be an observation sequence that is observable under the abstract preemptive semantics, but not under the abstract non-preemptive semantics. This observation sequence is $(\mathtt {T}1,(\mathsf {write}, \mathtt{x}), \ell _1),(\mathtt {T}2,(\mathsf {write}, \mathtt{x}), \ell _6),(\mathtt {T}1,(\mathsf {read}, \mathtt{y}), \ell _2),(\mathtt {T}1,\mathsf {else}, \ell _2),(\mathtt {T}1,(\mathsf {read}, \mathtt{x}), \ell _4),(\mathtt {T}1,\mathsf {then}, \ell _2),(\mathtt {T}1,(\mathsf {write}, \mathtt{dev}), \ell _5)$. The branch tagging records that the else branch is taken in $\ell _2$. The non-preemptive semantics cannot produce this observation sequences because it must also take the $\mathsf {else}$ branch in $\ell _2$ and can therefore not reach the $\mathsf {yield}$ statement and context-switch. As a site note, it is also not possible to transform this observation sequence into an equivalent one under the non-preemptive semantics because of the write to $\mathtt x$ at $\ell _6$ and the accesses to $\mathtt x$ in $\ell _1$ and $\ell _4$.

This example illustrates why branch tagging is crucial to soundness of the abstraction. If we assume a hypothetical abstract semantics without branch tagging we would get the following preemptive observation sequence: $(\mathtt {T}1,(\mathsf {write}, \mathtt{x}), \ell _1),(\mathtt {T}2,(\mathsf {write}, \mathtt{x}), \ell _6),(\mathtt {T}1,(\mathsf {read}, \mathtt{y}), \ell _2),(\mathtt {T}1,(\mathsf {read}, \mathtt{x}), \ell _4),(\mathtt {T}1,(\mathsf {write}, \mathtt{dev}), \ell _5)$. This sequence would also be a valid observation sequence under the non-preemptive semantics, because it could take the $\mathsf {then}$ branch in $\ell _2$ and reach the $\mathsf {yield}$ statement and context-switch.

##### Theorem 1

(soundness) Given concurrent program $\mathscr {C}$ and a synthesized program $\mathscr {C}'$ obtained by adding locks to $\mathscr {C}, [\![ \mathscr {C}'_{abs} ]\!]^{P} \Subset _{abs} [\![ \mathscr {C}_{abs} ]\!]^{\textit{NP}}\implies [\![ \mathscr {C}' ]\!]^{P} \Subset [\![ \mathscr {C} ]\!]^{\textit{NP}}$.

##### Proof

It is easier to prove the contrapositive: .

 means that there is an observation sequence $\omega '$ of $[\![ \mathscr {C}' ]\!]^{P}$ with no equivalent observation sequence in $[\![ \mathscr {C} ]\!]^{\textit{NP}}$. We now show that the abstract sequence $\omega '_{abs}$ in $[\![ \mathscr {C}'_{abs} ]\!]^{P}$ corresponding to the sequence $\omega '$ has no equivalent sequence in $[\![ \mathscr {C}_{abs} ]\!]^{\textit{NP}}$.

Towards contradiction we assume there is such an equivalent sequence $\omega _{abs}$ in $[\![ \mathscr {C}_{abs} ]\!]^{\textit{NP}}$. We show that if $\omega _{abs}$ indeed existed it would correspond to a concrete sequence $\omega$ that is equivalent to $\omega '$, thereby contradicting our assumption.

By (*A1*) $\omega _{abs}$ would have the same control flow as $\omega '_{abs}$ because of the branch tagging. By (*A2*) and (*A3*) $\omega _{abs}$ would have the same data-flow, meaning all reads from global variables are reading the values written by the same writes as in $\omega '_{abs}$. Since all interactions with the environment are abstracted to $\mathsf {write}(\mathtt {dev})$ the order of interactions must be the same between $\omega _{abs}$ and $\omega '_{abs}$. This means that, assuming all inputs and havocs are returning the same value, in the execution $\omega$ corresponding to $\omega _{abs}$ all variables valuation are identical to those in $\omega '$. Therefore, $\omega$ is feasible and its interaction with the environment is identical to $\omega '$ as all variable valuations are identical. Identical interaction with the environment is how equivalence between $\omega$ and $\omega '$ is defined. This concludes our proof. $\square$

#### Language inclusion modulo an independence relation

We define the problem of language inclusion modulo an independence relation. Let *I* be a non-reflexive, symmetric binary relation over an alphabet ${\varSigma }$. We refer to *I* as the *independence relation* and to elements of *I* as *independent* symbol pairs. We define a symmetric binary relation $\approx _I$ over words in ${\varSigma }^*$: for all words $\sigma , \sigma ' \in {\varSigma }^*$ and $(\alpha , \beta ) \in I, (\sigma \cdot \alpha \beta \cdot \sigma ', \sigma \cdot \beta \alpha \cdot \sigma ') \in \, \approx _I$. Let $\approx ^t_I$ denote the reflexive transitive closure of $\approx _I$.^2^ Given a language $\mathcal{L}$ over ${\varSigma }$, the closure of $\mathcal{L}$ w.r.t. *I*, denoted $\mathrm {Clo}_I(\mathcal{L})$, is the set $\{\sigma \in {\varSigma }^* {:}\ \exists \sigma ' \in \mathcal L \text { with } (\sigma ,\sigma ') \in \, \hbox { }\ \approx ^t_I\}$. Thus, $\mathrm {Clo}_I(\mathcal{L})$ consists of all words that can be obtained from some word in $\mathcal{L}$ by repeatedly commuting adjacent independent symbol pairs from *I*.

##### Definition 1

(*Language inclusion modulo an independence relation*) Given NFAs *A*, *B* over a common alphabet ${\varSigma }$ and an independence relation *I* over ${\varSigma }$, the language inclusion problem modulo *I* is: ${\mathscr {L}}(\text{ A }) \subseteq \mathrm {Clo}_I({\mathscr {L}}(\text{ B }))$?

#### Data independence relation

We define the data independence relation ${I_D}$ over our observable symbols. Two symbols $\alpha =( tid _\alpha ,a_\alpha ,\ell _\alpha )$ and $\beta =( tid _\beta ,a_\beta ,\ell _\beta )$ are independent, $(\alpha ,\beta )\in {I_D}$, iff (I0) $tid _\alpha \ne tid _\beta$ and one of the following hold:

1. $$a_\alpha $$ or $$a_\beta $$ in $$\{\mathsf {then},\mathsf {else},\mathsf {loop},\mathsf {loopexit}\} $$
2. $a_\alpha$ and $a_\beta$ are both $(\mathsf {read}, var )$
3. $a_\alpha$ is in $\{(\mathsf {write}, var _\alpha ),\ (\mathsf {read}, var _\alpha )\}$ and $a_\beta$ is in $\{(\mathsf {write}, var _\beta ),\ (\mathsf {read}, var _\beta )\}$ and $var _\alpha \ne var _\beta$

#### Checking preemption-safety

Under abstraction, we model each thread as a nondeterministic finite automaton (NFA) over a finite alphabet consisting of abstract observable symbols. This enables us to construct NFAs $\mathsf {NP}_{\textit{abs}}$ and ${\mathsf {P}}'_{\textit{abs}}$ accepting the languages $[\![ \mathscr {C}_{\textit{abs}} ]\!]^{\textit{NP}}$ and $[\![ \mathscr {C}_{\textit{abs}}' ]\!]^{P}$, respectively. $\mathscr {C}_{\textit{abs}}$ is the abstract program corresponding to the input program $\mathscr {C}$ and $\mathscr {C}_{\textit{abs}}'$ is the program corresponding to the result of the synthesis $\mathscr {C}'$. It turns out that preemption-safety of $\mathscr {C}'$ w.r.t. $\mathscr {C}$ is implied by preemption-safety of $\mathscr {C}_{\textit{abs}}'$ w.r.t. $\mathscr {C}_{\textit{abs}}$, which, in turn, is implied by *language inclusion modulo* ${I_D}$ of NFAs ${\mathsf {P}}'_{\textit{abs}}$ and $\mathsf {NP}_{\textit{abs}}$. NFAs ${\mathsf {P}}'_{\textit{abs}}$ and $\mathsf {NP}_{\textit{abs}}$ satisfy language inclusion modulo ${I_D}$ if any word accepted by ${\mathsf {P}}'_{\textit{abs}}$ is equivalent to some word obtainable by repeatedly commuting adjacent independent symbol pairs in a word accepted by $\mathsf {NP}_{\textit{abs}}$.

##### Proposition 1

Given concurrent programs $\mathscr {C}$ and $\mathscr {C}', [\![ \mathscr {C}'_{abs} ]\!]^{P} \Subset _{abs} [\![ \mathscr {C}_{abs} ]\!]^{\textit{NP}}$ iff ${\mathscr {L}}({\mathsf {P}}'_{\textit{abs}}) \subseteq \mathrm {Clo}_{{I_D}}({\mathscr {L}}(\mathsf {NP}_{\textit{abs}}))$.

##### Proof

By construction ${\mathsf {P}}'_{\textit{abs}}$, resp. $\mathsf {NP}_{\textit{abs}}$, accept exactly the observation sequences that $\mathscr {C}'_{abs}$, resp. $\mathscr {C}_{abs}$, may produce under the preemptive, resp. non-preemptive, semantics (denoted by $[\![ \mathscr {C}'_{abs} ]\!]^{P}$, resp. $[\![ \mathscr {C}_{abs} ]\!]^{\textit{NP}}$). It remains to show that two observation sequences $\omega _1=\alpha _0\ldots \alpha _k$ and $\omega _2=\beta _0\ldots \beta _k$ are equivalent iff $\omega _1\in \mathrm {Clo}_{{I_D}}(\{\omega _2\})$.

We first show that $\omega _1\in \mathrm {Clo}_{{I_D}}(\{\omega _2\})$ implies $\omega _1$ is equivalent to $\omega _2$. The proof proceeds by induction: The base case is that no symbols are swapped and is trivially true. The inductive case assumes that $\omega '$ is equivalent to $\omega _2$ and we needs to show that after one single swap operation in $\omega '$, resulting in $\omega '', \omega '$ is equivalent to $\omega ''$ and therefore by transitivity also equivalent to $\omega _2$. Rule (*A1*) holds because ${I_D}$ does not allow symbols of the same thread to be swapped (I0). To prove (*A2*) we use the fact that writes to the same variable cannot be swapped (*I2*), (*I3*). To prove (*A3*) we use the fact that reads and writes to the same variable are not independent (*I2*), (*I3*).

It remains to show that $\omega _1$ is equivalent to $\omega _2$ implies $\omega _1 \in \mathrm {Clo}_{{I_D}}(\{\omega _2\})$. Clearly $\omega _1$ and $\omega _2$ consist of the same multiset of symbols (*A1*). Therefore it is possible to transform $\omega _2$ into $\omega _1$ by swapping adjacent symbols. It remains to show that all swaps involve independent symbols. By (*A1*) the order of events in each thread does not change, therefore condition (I0) is always fulfilled. Branch tags can swap with every other symbol (*I1*) and accesses to different variables can swap with each other (*I3*). For each variables $ShVar$ (*A2*) ensures that writes are in the same order and (*A3*) allows reads in between to be reordered. These swaps are allowed by (*I2*). No other swaps can occur. $\square$

### Checking language inclusion

We first focus on the problem of language inclusion modulo an independence relation (Definition 1). This question corresponds to preemption-safety (Theorem 1, Proposition 1) and its solution drives our synchronization synthesis.

#### Theorem 2

For NFAs *A*, *B* over alphabet ${\varSigma }$ and a symmetric, irreflexive independence relation $I\subseteq {\varSigma }\times {\varSigma }$, the problem ${\mathscr {L}}(A)\subseteq \mathrm {Clo}_I({\mathscr {L}}(B))$ is undecidable [2].

We now show that this general undecidability result extends to our specific NFAs and independence relation ${I_D}$.

**Fig. 14:**
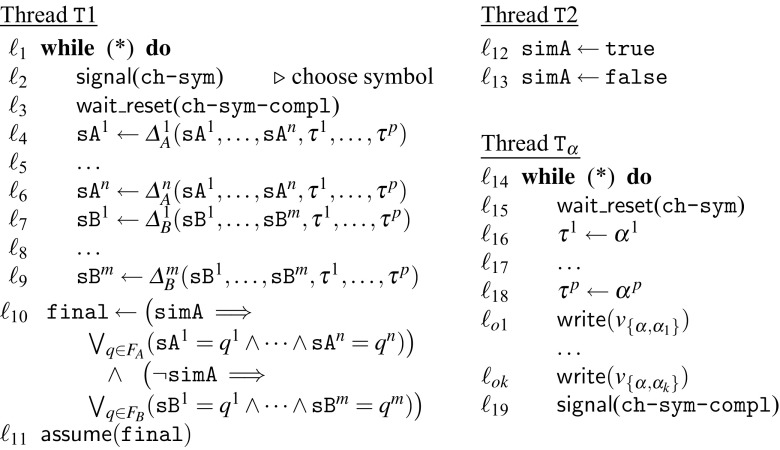
Simulator algorithm

#### Theorem 3

For NFAs ${\mathsf {P}}'_{\textit{abs}}$ and $\mathsf {NP}_{\textit{abs}}$ constructed from $\mathscr {C}_{\textit{abs}}$, the problem ${\mathscr {L}}({\mathsf {P}}'_{\textit{abs}})\subseteq \mathrm {Clo}_{{I_D}}({\mathscr {L}}(\mathsf {NP}_{\textit{abs}}))$ is undecidable.

#### Proof

Our proof is by reduction from the language inclusion modulo an independence relation problem (Definition 1). Theorem 3 follows from the undecidability of this problem (Theorem 2).

Assume we are given NFAs $A=(Q_A,{\varSigma },{\varDelta }_A,Q_{\iota ,A},F_A)$ and $B=(Q_B,{\varSigma },{\varDelta }_B,Q_{\iota ,B},F_B)$ and an independence relation $I\subseteq {\varSigma }\times {\varSigma }$. Without loss of generality we assume $A$ and $B$ to be deterministic, complete, and free of $\epsilon$-transitions, meaning from every state there is exactly one transition for each symbol. We show that we can construct a program $\mathscr {C}_{\textit{abs}}$ that is preemption-safe iff ${\mathscr {L}}(A)\subseteq \mathrm {Clo}_I({\mathscr {L}}(B))$.

For our reduction we construct a program $\mathscr {C}_{\textit{abs}}$ that simulates $A$ or $B$ if run with a preemptive scheduler and simulates only $B$ if run with a non-preemptive scheduler. Note that ${\mathscr {L}}(A)\cup {\mathscr {L}}(B)\subseteq \mathrm {Clo}_I({\mathscr {L}}(B))$ iff ${\mathscr {L}}(A)\subseteq \mathrm {Clo}_I({\mathscr {L}}(B))$. For every symbol $\alpha \in {\varSigma }$ our simulator produces a sequence $\omega _\alpha$ of abstract observable symbols. We say two such sequences $\omega _\alpha$ and $\omega _\beta$ *commute* if $\omega _\alpha \cdot \omega _\beta \approx ^t_{I_D}\omega _\beta \cdot \omega _\alpha$, i.e, if $\omega _\beta \cdot \omega _\alpha$ can be obtained from $\omega _\alpha \cdot \omega _\beta$ by repeatedly swapping adjacent symbol pairs in ${I_D}$.

We will show that (a) $\mathscr {C}_{\textit{abs}}$ simulates $A$ or $B$ if run with a preemptive scheduler and simulates only $B$ if run with a non-preemptive scheduler, and (b) sequences $\omega _\alpha$ and $\omega _\beta$ commute iff $(\alpha ,\beta )\in I$.

The simulator is shown in Fig. 14. States and symbols of $A$ and $B$ are mapped to natural numbers and represented as bitvectors to enable simulation using the language ${{\mathscr {W}}}_{\textit{abs}}$. In particular we use Boolean guard variables from ${{\mathscr {W}}}_{\textit{abs}}$ to represent the bitvectors. We use $\mathtt {true}$ to represent 1 and $\mathtt {false}$ to represent 0. As the state space and the alphabet are finite we know the number of bits needed a priori. We use *n*, *m*, and *p* for the number of bits needed to represent $Q_A, Q_B$, and ${\varSigma }$, respectively. The transition functions ${\varDelta }_A$ and ${\varDelta }_B$ likewise work on the individual bits. We represent bitvector *x* of length *n* as $x^1\ldots x^n$.

Thread $\mathtt {T}1$ simulates both automata *A* and *B* simultaneously. We assume the initial states of $A$ and $B$ are mapped to the number 0. In each iteration of the loop in thread $\mathtt {T}1$ a symbol $\alpha \in {\varSigma }$ is chosen non-deterministically and applied to both automata (we discuss this step in the next paragraph). Whether thread $\mathtt {T}1$ simulates $A$ or $B$ is decided only in the end: depending on the value of $\mathtt {simA}$ we assert that a final state of $A$ or $B$ was reached. The value of $\mathtt {simA}$ is assigned in thread $\mathtt {T}2$ and can only be $\mathtt {true}$ if $\mathtt {T}2$ is preempted between locations $\ell _{12}$ and $\ell _{13}$. With the non-preemptive scheduler the variable $\mathtt {simA}$ will always be $\mathtt {false}$ because thread $\mathtt {T}2$ cannot be preempted. The simulator can only reach the $\langle \mathtt {terminated}\rangle$ state if all assumptions hold as otherwise it would end in the $\langle \mathtt {failed}\rangle$ state. The guard $\mathtt {final}$ will only be assigned $\mathtt {true}$ in $\ell _{10}$ if either $\mathtt {simA}$ is $\mathtt {false}$ and a final state of $B$ has been reached or if $\mathtt {simA}$ is $\mathtt {true}$ and a final state of $A$ has been reached. Therefore the valid non-preemptive executions can only simulate $B$. In the preemptive setting the simulator can simulate either $A$ or $B$ because $\mathtt {simA}$ can be either $\mathtt {true}$ or $\mathtt {false}$. Note that the statement in location $\ell _{10}$ executes atomically and the value of $\mathtt {simA}$ cannot change during its evaluation. This means that ${\mathsf {P}}'_{\textit{abs}}$ simulates ${\mathscr {L}}(A)\cup {\mathscr {L}}(B)$ and $\mathsf {NP}_{\textit{abs}}$ simulates ${\mathscr {L}}(B)$.

We use $\tau$ to store the symbol used by the transition function. The choice of the next symbol needs to be non-deterministic to enable simulation of $A,B$ and there is no havoc statement in ${{\mathscr {W}}}_{\textit{abs}}$. We therefore use the fact that the next thread to execute is chosen non-deterministically at a preemption point. We define a thread $\mathtt {T}_\alpha$ for every $\alpha \in {\varSigma }$ that assigns to $\tau$ the number $\alpha$ maps to. Threads $\mathtt {T}_\alpha$ can only run if the conditional variable ch-sym is set to 1 by the $\mathsf {notify}$ statement in $\ell _{2}$. The  in $\ell _{3}$ is a preemption point for the non-preemptive semantics. Then, exactly one thread $\mathtt {T}_\alpha$ can proceed because the  statement in $\ell _{15}$ atomically resets ch-sym to 0. After setting $\tau$ and outputting the representation of $\alpha$ thread $\mathtt {T}_\alpha$, notifies thread $\mathtt {T}1$ using condition variable ch-sym-compl. Another symbol can only be produced in the next loop iteration of $\mathtt {T}1$.

To produce an observable sequence faithful to *I* for each symbol in ${\varSigma }$ we define a homomorphism *h* that maps symbols from ${\varSigma }$ to sequences of observables. Assuming the symbol $\alpha \in {\varSigma }$ is chosen, we produce the following observables:

- *Loop tag* To output $\alpha$ the thread $\mathtt {T}_\alpha$ has to perform one loop iteration. This implicitly produces a loop tag $(\mathtt {T}_\alpha , \mathsf {loop}, \ell _{14})$.
- *Conflict variables* For each pair of $(\alpha , \alpha _i) \notin I$, we define a conflict variable $v_{\{\alpha , \alpha _i\}}$. Note that $v_{\{\alpha , \alpha _i\}}=v_{\{\alpha _i, \alpha \}}$ and two writes to $v_{\{\alpha , \alpha _i\}}$ do not commute under ${I_D}$. For each $\alpha _i$, we produce a tag $(T_\alpha , (\mathsf {write},v_{\{\alpha , \alpha _i\}}, \ell _{oi}))$. Therefore if two variables $\alpha _1$ and $\alpha _2$ are dependent the observation sequences produced for each of them will contain a write to $v_{\{\alpha _1, \alpha _2\}}$.

Formally, the homomorphism *h* is given by $h(\alpha ) = (\mathtt {T}_\alpha , \mathsf {loop}, \ell _{14});(\mathtt {T}_\alpha , (\mathsf {write},v_{\{\alpha , \alpha _1\}}),\ell _{o1}); \cdots ; (\mathtt {T}_\alpha , (\mathsf {write},v_{\{\alpha , \alpha _k\}}), \ell _{ok})$. For a sequence $\sigma =\alpha _1\ldots \alpha _n$ use define $h(\sigma )=h(\alpha _1)\ldots h(\alpha _n)$.

We show that $(\alpha _1,\alpha _2)\in I$ iff $h(\alpha _1)$ and $h(\alpha _2)$ commute. The loop tags are independent iff $\alpha _1\ne \alpha _2$. If $\alpha _1=\alpha _2$ then $(\alpha _1,\alpha _2)\notin I$ and $h(\alpha _1)$ and $h(\alpha _2)$ do not commute due to the loop tags. Assuming $(\alpha _1,\alpha _2)\in I$ then $h(\alpha _1)$ and $h(\alpha _2)$ commute because they have no common conflict variable they write to. On the other hand, if $(\alpha _1,\alpha _2)\notin I$, then both $h(\alpha _1)$ and $h(\alpha _2)$ will contain $(\mathtt {T}_{\alpha _{\{1,2\}}}, (\mathsf {write},v_{\{\alpha _1, \alpha _2\}}), \ell _{oi})$ and therefore cannot commute. We extend this result to sequences and have that ${h(\sigma ')\approx ^t_{I_D}h(\sigma )}$ iff ${\sigma ' \approx ^t_I \sigma }$.

This concludes our reduction. It remains to show that $\mathscr {C}_{\textit{abs}}$ is preemption-safe iff ${\mathscr {L}}(A)\subseteq \mathrm {Clo}_I({\mathscr {L}}(B))$. By Proposition 1 it suffices to show that ${\mathscr {L}}(A)\subseteq \mathrm {Clo}_I({\mathscr {L}}(B))$ iff ${\mathscr {L}}({\mathsf {P}}'_{\textit{abs}})\subseteq \mathrm {Clo}_{{I_D}}({\mathscr {L}}(\mathsf {NP}_{\textit{abs}}))$.

1. We assume that ${\mathscr {L}}(A)\subseteq \mathrm {Clo}_I({\mathscr {L}}(B))$. Then, for every word $\sigma \in {\mathscr {L}}(A)$ we have that $\sigma \in \mathrm {Clo}_I({\mathscr {L}}(B))$. By construction $h(\sigma )\in {\mathscr {L}}({\mathsf {P}}'_{\textit{abs}})$. It remains to show that $h(\sigma )\in \mathrm {Clo}_{{I_D}}({\mathscr {L}}(\mathsf {NP}_{\textit{abs}}))$. By $\sigma \in \mathrm {Clo}_I({\mathscr {L}}(B))$ we know there exists a word $\sigma '\in {\mathscr {L}}(B)$, such that $\sigma '\approx _I^t \sigma$. Therefore also $h(\sigma ')\approx ^t_{I_D}h(\sigma )$ and by construction $h(\sigma ')\in {\mathscr {L}}(\mathsf {NP}_{\textit{abs}})$.
2. We assume that ${\mathscr {L}}(A)\nsubseteq \mathrm {Clo}_I({\mathscr {L}}(B))$. Then, there exists a word $\sigma \in {\mathscr {L}}(A)$ such that $\sigma \notin \mathrm {Clo}_I({\mathscr {L}}(B))$. By construction $h(\sigma )\in {\mathscr {L}}({\mathsf {P}}'_{\textit{abs}})$. Let us assume towards contradiction that $h(\sigma ) \in \mathrm {Clo}_{{I_D}}({\mathscr {L}}(\mathsf {NP}_{\textit{abs}}))$. Then there exists a word $\omega$ in ${\mathscr {L}}(\mathsf {NP}_{\textit{abs}})$ such that $\omega \approx ^t_{I_D}h(\sigma )$. By construction, this implies there exists some $\sigma '\in {\mathscr {L}}(B)$ such that $\omega = h(\sigma ')$ and $h(\sigma ') \approx ^t_{I_D}h(\sigma )$. Thus, there exists $\sigma '\in {\mathscr {L}}(B)$ such that $\sigma ' \approx ^t_I \sigma$. This implies $\sigma \in \mathrm {Clo}_I({\mathscr {L}}(B))$, which is a contradiction. $\square$

Fortunately, a bounded version of the language inclusion modulo *I* problem is decidable. Recall the relation $\approx _I$ over ${\varSigma }^*$ from Sect. 6.1. We define a symmetric binary relation $\approx _I^i$ over ${\varSigma }^*$: $(\sigma , \sigma ') \in \, \approx _I^i$ iff $\exists (\alpha ,\beta ) \in I$: $(\sigma , \sigma ') \in \, \approx _I, \sigma [i] = \sigma '[i+1] = \alpha$ and $\sigma [i+1] = \sigma '[i] = \beta$. Thus $\approx ^i_I$ consists of all words that can be obtained from each other by commuting the symbols at positions *i* and $i+1$. We next define a symmetric binary relation $\asymp$ over ${\varSigma }^*$: $(\sigma ,\sigma ') \in \, \asymp$ iff $\exists \sigma _1,\ldots ,\sigma _t$: $(\sigma ,\sigma _1) \in \, \approx _I^{i_1},\ldots , (\sigma _{t},\sigma ') \in \, \approx _I^{i_{t+1}}$ and $i_1< \ldots < i_{t+1}$. The relation $\asymp$ intuitively consists of words obtained from each other by making a single forward pass commuting multiple pairs of adjacent symbols. We recursively define $\asymp ^k$ as follows: $\asymp ^0$ is the identity relation *id*. For $k>0$ we define $\asymp ^k = \asymp \circ \asymp ^{k-1}$, the composition of $\asymp$ with $\asymp ^{k-1}$. Given a language $\mathcal{L}$ over ${\varSigma }$, we use $\mathrm {Clo}_{k,I}(\mathcal{L})$ to denote the set $\{\sigma \in {\varSigma }^*: \exists \sigma ' \in \mathcal L \text { with } (\sigma ,\sigma ') \in \, \asymp ^{\hbox { }\ \scriptstyle k}\}$. In other words, $\mathrm {Clo}_{k,I}(\mathcal{L})$ consists of all words which can be generated from $\mathcal{L}$ using a finite-state transducer that remembers at most *k* symbols of its input words in its states. By definition we have $\mathrm {Clo}_{0,I}(\mathcal L) = \mathcal L$.

#### Example 1

We assume the language $\mathcal{L}=\{a,b\}^*$, where $(a,b)\in I$.

- $\textit{aaab} \asymp _I^1 \textit{aaba}$ because one can swap the letters as position 3 and 4.
- $\textit{aaab} \not \asymp _I^1 \textit{abaa}$ because one can only swap the letters as position 3 and 4 in one pass, but not after that swap 2 and 3.
- However, $\textit{aaab} \asymp _I^2 \textit{abaa}$, as two passes suffice to do the two swaps.
- $\textit{baaa} \asymp _I^1 \textit{aaba}$ because in a single pass one can swap 1 and 2 and then 2 and 3.

#### Definition 2

(*Bounded language inclusion modulo an independence relation*) Given NFAs $A, B$ over ${\varSigma }, I\subseteq {\varSigma }\times {\varSigma }$ and a constant $k\ge 0$, the *k*-bounded language inclusion problem modulo *I* is: ${\mathscr {L}}(\text{ A })\subseteq \mathrm {Clo}_{k,I}({\mathscr {L}}(\text{ B }))$?

#### Theorem 4

For NFAs $A, B$ over ${\varSigma }, I\subseteq {\varSigma }\times {\varSigma }$ and a constant $k\ge 0, {\mathscr {L}}(\text{ A }) \subseteq \mathrm {Clo}_{k,I}({\mathscr {L}}(\text{ B }))$ is decidable.

We present an algorithm to check *k*-bounded language inclusion modulo *I*, based on the antichain algorithm for standard language inclusion [11].

### Antichain algorithm for language inclusion

Given a partial order $(X, \sqsubseteq )$, an antichain over *X* is a set of elements of *X* that are incomparable w.r.t. $\sqsubseteq$. In order to check ${\mathscr {L}}(A)\subseteq {\mathscr {L}}(B)$ for NFAs $A = (Q_A,{\varSigma },{\varDelta }_A,Q_{\iota ,A},F_A)$ and $B = (Q_B,{\varSigma },{\varDelta }_B,Q_{\iota ,B},F_B)$, the antichain algorithm proceeds by exploring $A$ and $B$ in lockstep. Without loss of generality we assume that $A$ and $B$ do not have $\epsilon$-transitions. While $A$ is explored nondeterministically, $B$ is determinized on the fly for exploration. The algorithm maintains an antichain, consisting of tuples of the form $(s_A, S_B)$, where $s_A\in Q_A$ and $S_B\subseteq Q_B$. The ordering relation $\sqsubseteq$ is given by $(s_A, S_B) \sqsubseteq (s'_A, S'_B)$ iff $s_A= s'_A$ and $S_B\subseteq S'_B$. The algorithm also maintains a *frontier* set of tuples *yet* to be explored.

Given state $s_A\in Q_A$ and a symbol $\alpha \in {\varSigma }$, let $\mathsf{succ}_\alpha (s_A)$ denote $\{s_A' \in Q_A: (s_A,\alpha ,s_A') \in {\varDelta }_A\}$. Given set of states $S_B\subseteq Q_B$, let $\mathsf{succ}_\alpha (S_B)$ denote $\{s_B'\in Q_B: \exists s_B\in S_B:\ (s_B,\alpha ,s_B')\in {\varDelta }_B\}$. Given tuple $(s_A, S_B)$ in the frontier set, let $\mathsf{succ}_\alpha (s_A, S_B)$ denote $\{(s'_A,S'_B): s'_A\in \mathsf{succ}_\alpha (s_A), S'_B= \mathsf{succ}_\alpha (S_B)\}$.

In each step, the antichain algorithm explores $A$ and $B$ by computing $\alpha$-successors of all tuples in its current frontier set for all possible symbols $\alpha \in {\varSigma }$. Whenever a tuple $(s_A, S_B)$ is found with $s_A\in F_A$ and $S_B\cap F_B=\emptyset$, the algorithm reports a counterexample to language inclusion. Otherwise, the algorithm updates its frontier set and antichain to include the newly computed successors using the two rules enumerated below. Given a newly computed successor tuple $p'$, if there does not exist a tuple *p* in the antichain with $p \sqsubseteq p'$, then $p'$ is added to the frontier set or antichain (*Rule R1*). If $p'$ is added and there exist tuples $p_1, \ldots , p_n$ in the antichain with $p' \sqsubseteq p_1, \ldots , p_n$, then $p_1, \ldots , p_n$ are removed from the antichain (*Rule R2*). The algorithm terminates by either reporting a counterexample, or by declaring success when the frontier becomes empty.

### Antichain algorithm for *k*-bounded language inclusion modulo *I*

This algorithm is essentially the same as the standard antichain algorithm, with the automaton $B$ above replaced by an automaton $B_{k,I}$ accepting $\mathrm {Clo}_{k,I}({\mathscr {L}}(\text{ B }))$. The set $Q_{B_{k,I}}$ of states of $B_{k,I}$ consists of triples $(s_B, \eta _1, \eta _2)$, where $s_B\in Q_B$ and $\eta _1, \eta _2$ are words over ${\varSigma }$ of up to *k* length. Intuitively, the words $\eta _1$ and $\eta _2$ store symbols that are expected to be matched later along a run. The word $\eta _1$ contains a list of symbols for transitions taken by $B_{k,I}$, but not yet matched in $B$, whereas $\eta _2$ contains a list of symbols for transitions taken in $B$, but not yet matched in $B_{k,I}$. We use $\emptyset$ to denote the empty list. Since for every transition of $B_{k,I}$, the automaton $B$ will perform one transition, we have $|\eta _1| = |\eta _2|$. The set of initial states of $B_{k,I}$ is $\{(s_B,\emptyset ,\emptyset ): s_B\in Q_{\iota ,B}\}$. The set of final states of $B_{k,I}$ is $\{(s_B,\emptyset ,\emptyset ): s_B\in F_B\}$. The transition relation ${\varDelta }_{B_{k,I}}$ is constructed by repeatedly performing the following steps, in order, for each state $(s_B, \eta _1, \eta _2)$ and each symbol $\alpha$. In what follows, $\eta [\setminus i]$ denotes the word obtained from $\eta$ by removing its *i*th symbol.

Given $$(s_B,\eta _1,\eta _2)$$ and $$\alpha $$

- *Step S1* Pick *new* $s'_B$ and $\beta \in {\varSigma }$ such that $(s_B, \beta , s_B') \in {\varDelta }_B$
- *Step S2*
  - If $\forall i$: $\eta _1[i] \ne \alpha$ and $\alpha$ is independent of all symbols in $\eta _1$, $\eta _2' \, \mathtt {:=}\, \eta _2\cdot \alpha$ and $\eta _1' \, \mathtt {:=}\, \eta _1$,
  - else, if $\exists i$: $\eta _1[i] = \alpha$ and $\alpha$ is independent of all symbols in $\eta _1$ prior to $i, \eta _1' \, \mathtt {:=}\, \eta _1[\setminus i]$ and $\eta _2'\, \mathtt {:=}\, \eta _2$
  - else, go to S1
- *Step S3*
  - If $\forall i$: $\eta _2'[i] \ne \beta$ and $\beta$ is independent of all symbols in $\eta _2', \eta _1'' \, \mathtt {:=}\eta _1' \,\cdot \beta$ and $\eta _2''\,\mathtt {:=}\,\eta _2'$,
  - else, if $\exists i$: $\eta _2'[i] = \beta$ and $\beta$ is independent of all symbols in $\eta _2'$ prior to $i, \eta _2' \, \mathtt {:=}\, \eta _2'[\setminus i]$ and $\eta _1''\,\mathtt {:=}\, \eta _1'$
  - else, go to S1
- *Step S4* Add $((s_B, \eta _1, \eta _2),\alpha ,(s'_B, \eta _1'', \eta _2''))$ to ${\varDelta }_{B_{k,I}}$ and go to 1.

#### Example 2

**Fig. 15:**
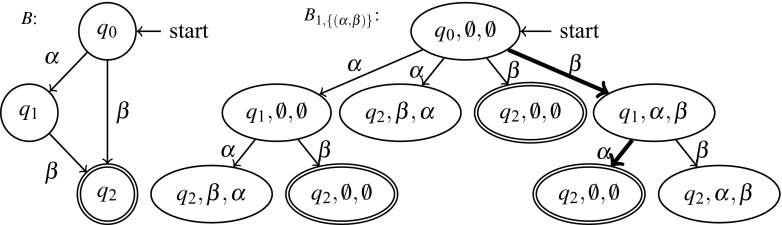
Example for illustrating construction of $B_{k,I}$ for $k = 1$ and $I = \{(\alpha , \beta )\}$

In Fig. 15, we have an NFA $B$ with ${\mathscr {L}}(\text{ B })= \{\alpha \beta , \beta \}, I = \{(\alpha ,\beta )\}$ and $k = 1$. The states of $B_{k,I}$ are triples $(q, \eta _1, \eta _2)$, where $q \in Q_B$ and $\eta _1, \eta _2\in \{\alpha ,\beta \}^*$. We explain the derivation of a couple of transitions of $B_{k,I}$. The transition shown in bold from $(q_0, \emptyset ,\emptyset )$ on symbol $\beta$ is obtained by applying the following steps once: S1. Pick $q_1$ following the transition $(q_0, \alpha , q_1) \in {\varDelta }_B$. S2(a). $\eta _2'\ \mathtt {:=}\ \beta , \eta _1'\ \mathtt {:=}\ \emptyset$. S3(a). $\eta _1''\ \mathtt {:=}\ \alpha , \eta _2''\ \mathtt {:=}\ \beta$. S4. Add $((q_0, \emptyset , \emptyset ),\beta ,(q_1, \alpha , \beta ))$ to ${\varDelta }_{B_{k,I}}$. The transition shown in bold from $(q_1, \alpha ,\beta )$ on symbol $\alpha$ is obtained as follows: S1. Pick $q_2$ following the transition $(q_1, \beta , q_2) \in {\varDelta }_B$. S2(b). $\eta _1'\ \mathtt {:=}\ \emptyset , \eta _2'\ \mathtt {:=}\ \beta$. S3(b). $\eta _2''\ \mathtt {:=}\ \emptyset , \eta _1''\ \mathtt {:=}\ \emptyset$. S4. Add $((q_1, \alpha , \beta ),\beta ,(q_2, \emptyset , \emptyset ))$ to ${\varDelta }_{B_{k,I}}$. It can be seen that $B_{k,I}$ accepts the language $\{\alpha \beta ,\beta \alpha ,\beta \} = \mathrm {Clo}_{k,I}({\mathscr {L}}(\text{ B }))$.

#### Proposition 2

Given $k\ge 0$, the automaton $B_{k,I}$ accepts at least $\mathrm {Clo}_{k,I}({\mathscr {L}}(\text{ B }))$.

#### Proof

The proof is by induction on *k*. The base case is trivially true, as ${\mathscr {L}}(B_{0,I})={\mathscr {L}}(B)=\mathrm {Clo}_{0,I}({\mathscr {L}}(B))$. The induction case assumes that $B_{k,I}$ accepts at least $\mathrm {Clo}_{k,I}({\mathscr {L}}(\text{ B }))$ and we want to show that $B_{k+1,I}$ accepts at least $\mathrm {Clo}_{k+1,I}({\mathscr {L}}(B))$. We take a word $\omega \in \mathrm {Clo}_{k+1,I}({\mathscr {L}}(B))$. It must be derived from a word $\omega '\in \mathrm {Clo}_{k,I}({\mathscr {L}}(B))$ by one additional forward pass of swapping. $B_{k+1,I}$ accepts $\omega$: In step S1 we pick the same transitions in ${\varDelta }_B$ as to accept $\omega '$. Steps S2 and S3 will be identical as for $\omega '$ with the exception of those adjacent symbol pairs that are newly swapped in $\omega$. For those pairs the symbols are first added to $\eta _2$ and $\eta _1$ by S2 and S3. In the next step they are removed because the swapping only allows adjacent symbols to be swapped. This also shows that the bound $k+1$ suffices to accept $\omega$. $\square$

In general NFA $B_{k,I}$ can accept words not in $\mathrm {Clo}_{k,I}({\mathscr {L}}(\text{ B }))$. Intuitively this is because $B_{k,I}$ has two stacks and can also accept words where the swapping is done in a backward pass (instead of a forward pass required in our definition). For our purposes it is sound to accept more words as long as they are obtained only by swapping independent symbols.

#### Proposition 3

Given $k\ge 0$, the automaton $B_{k,I}$ accepts at most $\mathrm {Clo}_I({\mathscr {L}}(B))$.

#### Proof

We need to show that $\omega '\in B_{k,I}\implies \omega '\in \mathrm {Clo}_I({\mathscr {L}}(B))$. For this we need to show that $\omega '$ is a permutation of a word $\omega \in {\mathscr {L}}(B)$ by repeatedly swapping independent, adjacent symbols. The word $\omega '$ must be a permutation of $\omega$ because $B_{k,I}$ only accepts if $\eta _1$ and $\eta _2$ are empty and the stacks represent exactly the symbols not matched yet in NFA $B$. Further, we need to show only independent symbols may be swapped. The stack $\eta _1$ contains the symbols not yet matched by $B$ and $\eta _2$ the symbols that were instead accepted by $B$, but not yet presented as input to $B_{k,I}$. Before adding a new symbol to the stack we ensure it is independent with all symbols on the other stack because once matched later it will have to come after all of these. When a symbols is removed it is ensured that it is independent with all symbols on its own stack because it is practically moved ahead of the other symbols on the stack. $\square$

### Language inclusion check algorithm

We develop a procedure to check language inclusion modulo *I* (Sect. 6.4) by iteratively increasing the bound *k*. The procedure is *incremental*: the check for $k+1$-bounded language inclusion modulo *I* only explores paths along which the bound *k* was exceeded in the previous iteration.

The algorithm for *k*-bounded language inclusion modulo *I* is presented as function Inclusion in Algorithm 1 (ignore Lines 22–25 for now). The antichain set consists of tuples of the form $(s_A, S_{B_{k,I}})$, where $s_A\in Q_A$ and $S_{B_{k,I}}\subseteq Q_B\times {\varSigma }^k \times {\varSigma }^k$. The frontier consists of tuples of the form $(s_A, S_{B_{k,I}},\textit{cex})$, where $\textit{cex}\in {\varSigma }^*$. The word $\textit{cex}$ is a sequence of symbols of transitions explored in $A$ to get to state $s_A$. If the language inclusion check fails, $\textit{cex}$ is returned as a counterexample to language inclusion modulo *I*. Each tuple in the frontier set is first checked for equivalence w.r.t. acceptance (Line 18). If this check fails, the function reports language inclusion failure and returns the counterexample $\textit{cex}$ (Line 18). If this check succeeds, the successors are computed (Line 20). If a successor satisfies rule R1, it is ignored (Line 21), otherwise it is added to the frontier (Line 26) and the antichain (Line 27). When adding a successor to the frontier the symbol $\alpha$ it appended to the counterexample, denoted as $\textit{cex}\cdot \alpha$. During the update of the antichain the algorithm ensures that its invariant is preserved according to rule R2.
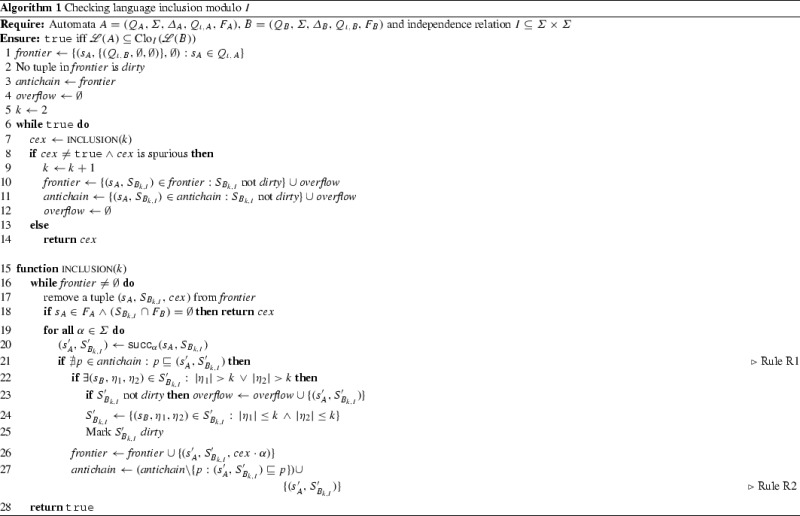


We need to ensure that our language inclusion honors the bound *k* by ignoring states that exceed the bound. These states are stored for later to allow for a restart of the language inclusion algorithm with a higher bound. Given a newly computed successor $(s_A', S_{B_{k,I}}')$ for an iteration with bound *k*, if there exists some $(s_B,\eta _1,\eta _2)$ in $S_{B_{k,I}}'$ such that the length of $\eta _1$ or $\eta _2$ exceeds *k* (Line 22), we remember the tuple $(s_A', S_{B_{k,I}}')$ in the set $overflow$ (Line 23). We then prune $S_{B_{k,I}}'$ by removing all states $(s_B,\eta _1,\eta _2)$ where $|\eta _1|> k \vee |\eta _2| > k$ (line 24) and mark $S_{B_{k,I}}'$ as *dirty* (line 24). If we find a counterexample to language inclusion we return it and test if it is spurious (Line 8). In case it is spurious we increase the bound to $k+1$, remove all dirty items from the antichain and frontier (lines 10–11), and add the items from the overflow set (Line 12) to the antichain set and frontier. Intuitively this will undo all exploration from the point(s) the bound was exceeded and restarts from that/those point(s).

We call a counterexample $\textit{cex}$ from our language inclusion procedure spurious if it is not a counterexample to the unbounded language inclusion, formally $\textit{cex}\in \mathrm {Clo}_I({\mathscr {L}}(B))$. This test is decidable because there is only a finite number of permutations of $\textit{cex}$. This spuriousness arises from the fact that the bounded language-inclusion algorithm is incomplete and every spurious example can be eliminated by sufficiently increasing the bound *k*. Note, however, that there exists automata and independence relations for which there is a (different) spurious counterexample for every *k*. In practice we test if a $\textit{cex}$ is spurious by building an automata $A$ that accepts exactly $\textit{cex}$ and running the language inclusion algorithm with *k* being the length of $\textit{cex}$. This is very fast because there is exactly one path through $A$.

#### Theorem 5

(bounded language inclusion check) The procedure inclusion of Algorithm 1 decides ${\mathscr {L}}(A)\subseteq {\mathscr {L}}(B_{k,I})$ for NFAs $A, B$, bound *k*, and independence relation *I*.

#### Proof

Our algorithm takes as arguments automata $A$ and $B$. Conceptually, the algorithm constructs $B_{k,I}$ and uses the antichain algorithm [11] to decide the language inclusion. For efficiency, we modify the original antichain language inclusion algorithm to construct the automaton $B_{I}$ on the fly in the successor relation $\mathsf{succ}$ (line 20). The bound *k* is enforced separately in line 22. $\square$

#### Theorem 6

(preemption-safety problem) If program $\mathscr {C}$ is not preemption-safe (), then Algorithm 1 will return $\mathtt {false}$.

#### Proof

By Theorem 1 we know . From Proposition 1 we get ${\mathscr {L}}({\mathsf {P}}_{abs}) \nsubseteq \mathrm {Clo}_{{I_D}}({\mathscr {L}}(\mathsf {NP}_{\textit{abs}}))$. From Proposition 3 we know that for any *k* this is equivalent to ${\mathscr {L}}({\mathsf {P}}_{abs}) \nsubseteq {\mathscr {L}}(B_{k,I})$, where $B=\mathsf {NP}_{\textit{abs}}$. Theorem 5 shows that Algorithm 1 decides this for any bound *k*. $\square$

## Finding and Enforcing Mutex Constraints in $\varvec{{\mathsf {P}}'_{\textit{abs}}}$

If the language inclusion check fails it returns a counterexample trace. Using this counterexample we derive a set of *mutual exclusion (mutex) constraints* that we enforce in ${\mathsf {P}}'_{\textit{abs}}$ to eliminate the counterexample and then rerun the language inclusion check with the new ${\mathsf {P}}'_{\textit{abs}}$.

### Finding mutex constraints

The counterexample $\textit{cex}$ returned by the language inclusion check is a sequence of observables. Since our observables record every branching decision it is easy to reconstruct from $\textit{cex}$ a sequence of event identifiers: $tid _0.\ell _0 ; \ldots ; tid _n.\ell _n$, where each $\ell _i$ is a location identifier from $\mathscr {C}_{\textit{abs}}$. In this section we use $\textit{cex}$ to refer to such sequences of event identifiers. We define the *neighborhood* of $\textit{cex}$, denoted $\textit{nhood}(\textit{cex})$, as the set of all traces that are permutations of the events in $\textit{cex}$ and preserve the order of events from the same thread. We separate traces in $\textit{nhood}(\textit{cex})$ into *good* and *bad traces*. Good traces are all traces that are infeasible under the non-preemptive semantics or that produce an observation sequence that is equivalent to that of a trace feasible under the non-preemptive semantics. All remaining traces in $\textit{nhood}(\textit{cex})$ are bad. The goal of our counterexample analysis is to characterize all bad traces in $\textit{nhood}(\textit{cex})$ in order to enable inference of mutex constraints.

In order to succinctly represent subsets of $\textit{nhood}(\textit{cex})$, we use *ordering constraints* between events expressed as *happens-before formulas* (*HB-formulas*) [15]. Intuitively, ordering constraints are of the following forms: (a) atomic ordering constraints $\varphi = A < B$ where *A* and *B* are events from $\textit{cex}$. The constraint $A < B$ represents the set of traces in $\textit{nhood}(\textit{cex})$ where event *A* is scheduled before event *B*; (b) Boolean combinations of atomic constraints $\varphi _1 \wedge \varphi _2, \varphi _1 \vee \varphi _2$ and $\lnot \varphi _1$. We have that $\varphi _1 \wedge \varphi _2$ and $\varphi _1 \vee \varphi _2$ respectively represent the intersection and union of the set of traces represented by $\varphi _1$ and $\varphi _2$, and that $\lnot \varphi _1$ represents the complement (with respect to $\textit{nhood}(\textit{cex})$) of the traces represented by $\varphi _1$.

#### Non-preemptive neighborhood

First, we define function ${\varPhi }$ to extract a conjunction of atomic ordering constraints from a trace $\pi$, such that all traces $\pi '$ in ${\varPhi }(\pi )$ produce an observation sequence equivalent to $\pi$. Then, we obtain a correctness constraint $\varphi$ that represents all good traces in $\textit{nhood}(\textit{cex})$. Remember, that the good traces are those that are observationally equivalent to a non-preemptive trace. The correctness constraint $\varphi$ is a disjunction over the ordering constraints from all traces in $\textit{nhood}(\textit{cex})$ that are feasible under non-preemptive semantics: $\varphi _G =\bigvee _{\pi \in {\text {non-preemptive}}} {\varPhi }(\pi )$.

${\varPhi }(\pi )$ enforces the order between conflicting accesses in the abstract trace $\pi$:

$$\begin{aligned} {\varPhi }(\pi ) = \bigwedge \{\mathtt {T}i.\ell _{j} <&\mathtt {T}k.\ell _{l} :~ i \ne k \wedge \mathtt {T}i.\ell _{j}~ \text{ precedes }~ \mathtt {T}k.\ell _{l}~ \text{ in }~ \pi \wedge \\&\mathtt {T}i.\ell _{j},\mathtt {T}k.\ell _{l}~ \text{ access } \text{ same } \text{ variable } \wedge \mathtt {T}i.\ell _{j}~\text{ or }~\mathtt {T}k.\ell _{l}~\text{ is } \text{ a } \text{ write }\} \end{aligned}$$

##### Example

Recall the counterexample trace from the running example in Sect. 3: $\textit{cex}= \mathtt {T}1.\ell _{1a};\ \mathtt {T}1.\ell _{1b};\ \mathtt {T}2.\ell _{1a};\ \mathtt {T}2.\ell _{1b};\ \mathtt {T}1.\ell _2;\ \mathtt {T}2.\ell _2;\ \mathtt {T}2.\ell _{3a};\ \mathtt {T}2.\ell _{3b};\ \mathtt {T}2.\ell _4;\ \mathtt {T}1.\ell _{3a};\ \mathtt {T}1.\ell _{3b};\ \mathtt {T}1.\ell _4$. There are two traces in $\textit{nhood}(\textit{cex})$ that are feasible under non-preemptive semantics:

- $$\pi _1=\mathtt {T}1.\ell _{1a};\ \mathtt {T}1.\ell _{1b};\ \mathtt {T}1.\ell _2;\ \mathtt {T}1.\ell _{3a};\ \mathtt {T}1.\ell _{3b};\ \mathtt {T}1.\ell _4;\ \mathtt {T}2.\ell _{1a};\ \mathtt {T}2.\ell _{1b};\ \mathtt {T}2.\ell _2;\ \mathtt {T}2.\ell _{3a};\ \mathtt {T}2.\ell _{3b};\ \mathtt {T}2.\ell _4$$ and
- $$\pi _2=\mathtt {T}2.\ell _{1a};\ \mathtt {T}2.\ell _{1b};\ \mathtt {T}2.\ell _2;\ \mathtt {T}2.\ell _{3a};\ \mathtt {T}2.\ell _{3b};\ \mathtt {T}2.\ell _4;\ \mathtt {T}1.\ell _{1a};\ \mathtt {T}1.\ell _{1b};\ \mathtt {T}1.\ell _2;\ \mathtt {T}1.\ell _{3a};\ \mathtt {T}1.\ell _{3b};\ \mathtt {T}1.\ell _4$$.

We represent

- $$\pi _1$$ as $${\varPhi }(\pi _1)= (\{\mathtt {T}1.\ell _{1a},\mathtt {T}1.\ell _{3a},\mathtt {T}1.\ell _{3b}\}<\mathtt {T}2.\ell _{3b}) \; \wedge \; (\mathtt {T}1.\ell _{3b}<\{\mathtt {T}2.\ell _{1a},\mathtt {T}2.\ell _{3a},\mathtt {T}2.\ell _{3b}\}) \; \wedge \; (\mathtt {T}1.\ell _{2}<\mathtt {T}2.\ell _{2}$$) and
- $$\pi _2$$ as $${\varPhi }(\pi _2)= (\mathtt {T}2.\ell _{3b}<\{\mathtt {T}1.\ell _{1a},\mathtt {T}1.\ell _{3a},\mathtt {T}1.\ell _{3b}\}) \; \wedge \; (\{\mathtt {T}2.\ell _{1a},\mathtt {T}2.\ell _{3a},\mathtt {T}2.\ell _{3b}\}<\mathtt {T}1.\ell _{3b}) \; \wedge \; (\mathtt {T}2.\ell _{2}<\mathtt {T}1.\ell _{2}$$).

The correctness specification is $\varphi _G = {\varPhi }(\pi _1) \vee {\varPhi }(\pi _2)$.

#### Counterexample enumeration and generalization

We next build a quantifier-free first-order formula ${\varPsi }_B$ over the event identifiers in $\textit{cex}$ such that any model of ${\varPsi }_B$ corresponds to a bad, feasible trace in $\textit{nhood}(\textit{cex})$. A trace is feasible if it respects the preexisting synchronization, which is not abstracted away. Bad traces are those that are feasible under the preemptive semantics and not in $\varphi _G$. Further, we define a generalization function *G* that works on conjunctions of atomic ordering constraints $\varphi$ by iteratively removing a constraint as long as the intersection of traces represented by $G(\varphi )$ and $\varphi _G$ is empty. This results in a local minimum of atomic ordering constraints in $G(\varphi )$, so that removing any remaining constraint would include a good trace in $G(\varphi )$. We iteratively enumerate models $\psi$ of ${\varPsi }_B$, building a constraint $\varphi _{B'}= {\varPhi }(\psi )$ for each model $\psi$ and generalizing $\varphi _{B'}$ to represent a larger set of bad traces using *G*. This results in an ordering constraint in disjunctive normal form $\varphi _B=\bigvee _{\psi \in {{\varPsi }_B}} G({\varPhi }(\psi ))$, such that the intersection of $\varphi _B$ and $\varphi _G$ is empty and the union equals $\textit{nhood}(\textit{cex})$.
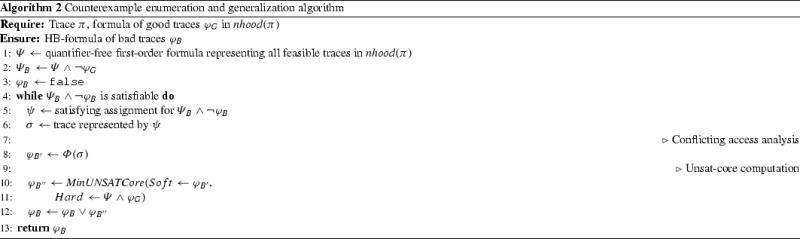


Algorithm 2 shows how the algorithm works. For each model $\psi$ of ${\varPsi }_B$ a trace $\sigma$ is extracted in Line 6. From the trace the formula $\varphi _{B'}$ is extracted using ${\varPhi }$ described above (Line 8). Line 10 describes the generalization function *G*, which is implemented using an unsat core computation. We construct a formula $\varphi _{B'} \wedge {\varPsi }\wedge \varphi _G$, where ${\varPsi }\wedge \varphi _G$ is a hard constraint and $\varphi _B'$ are soft constraints. A satisfying assignment to this formula models feasible traces that are observationally equivalent to a non-preemptive trace. Since $\sigma$ is a bad trace the formula $\varphi _{B'} \wedge {\varPsi }\wedge \varphi _G$ must be unsatisfiable. The result of the unsat core computation is a formula $\varphi _{B''}$ that is a conjunction of a minimal set of happens-before constraints required to ensure all trace represented by $\varphi _{B''}$ are bad.

##### Example

Our trace $\textit{cex}$ from Sect. 3 is generalized to $G({\varPhi }(cex))=\mathtt {T}2.\ell _{1a}<\mathtt {T}1.\ell _{3b} \wedge \mathtt {T}1.\ell _{3b}<\mathtt {T}2.\ell _{3b}$. This constraint captures the interleavings where $\mathtt {T}2$ interrupts $\mathtt {T}1$ between locations $\ell _{1a}$ and $\ell _{3b}$. Any trace that fulfills this constraint is bad. All bad traces in $\textit{nhood}(\textit{cex})$ are represented as $\varphi _B = (\mathtt {T}2.\ell _{1a}<\mathtt {T}1.\ell _{3b} \wedge \mathtt {T}1.\ell _{3b}<\mathtt {T}2.\ell _{3b})\, \vee \, (\mathtt {T}1.\ell _{1a}<\mathtt {T}2.\ell _{3b} \wedge \mathtt {T}2.\ell _{3b}<\mathtt {T}1.\ell _{3b})$.

#### Inferring mutex constraints

From each clause $\varphi$ in $\varphi _B$ described above, we infer mutex constraints to eliminate all bad traces satisfying $\varphi$. The key observation we exploit is that atomicity violations show up in our formulas as two simple patterns of ordering constraints between events.

**Fig. 16:**
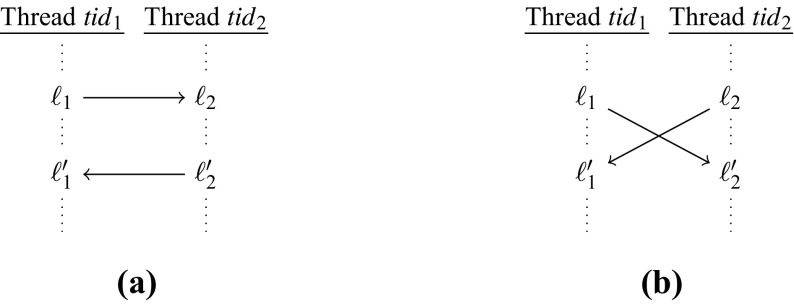
Atomicity violation patterns

1. The first pattern $$ tid _1.\ell _1< tid _2.\ell _2 \; \wedge \; tid _2.\ell '_2 < tid _1.\ell '_1$$ (visualized in Fig. 16a) indicates an atomicity violation (thread $$ tid _2$$ interrupts $$ tid _1$$ at a critical moment).
2. The second pattern is $$ tid _1.\ell _1< tid _2.\ell '_2 \; \wedge \; tid _2.\ell _2 < tid _1.\ell '_1$$ (visualized in Fig. 16b). This pattern is a generalization of the first pattern in that either $$ tid _1$$ interrupts $$ tid _2$$ or the other way round.

For both patterns the corresponding mutex constraint is $\mathrm {mtx}( tid _1.[\ell _1{:}\ell '_1], tid _2.[\ell _2{:}\ell '_2])$.

##### Example

The generalized counterexample constraint $\mathtt {T}2.\ell _{1a}<\mathtt {T}1.\ell _{3b} \wedge \mathtt {T}1.\ell _{3b}<\mathtt {T}2.\ell _{3b}$ yields the constraint mutex $\mathrm {mtx}(\mathtt {T}2.[\ell _{1a}{:}\ell _{3b}],\mathtt {T}1.[\ell _{3b}{:}\ell _{3b}])$. In the next section we show how this mutex constraint is enforced in ${\mathsf {P}}'_{\textit{abs}}$.

### Enforcing mutex constraints

To enforce mutex constraints in ${\mathsf {P}}'_{\textit{abs}}$, we prune paths in ${\mathsf {P}}'_{\textit{abs}}$ that violate the mutex constraints.

#### Conflicts

Given a mutex constraint $\mathrm {mtx}( tid _i.[\ell _1{:}\ell '_1], tid _j.[\ell _2{:}\ell '_2])$, a *conflict* is a tuple $(\ell _i^{\text {pre}},\ell _i^{\text {mid}},\ell _i^{\text {post}},\ell _j^{\text {cpre}},\ell _j^{\text {cpost}})$ of location identifiers satisfying the following:

- $$\ell _i^{\text {pre}}, \ell _i^{\text {mid}}, \ell _i^{\text {post}}$$ are adjacent locations in thread $$ tid _i$$,
- $$\ell _j^{\text {cpre}}, \ell _j^{\text {cpost}}$$ are adjacent locations in the other thread $$ tid _{j}$$,
- $$\ell _1 \le \ell _i^{\text {pre}}, \ell _i^{\text {mid}}, \ell _i^{\text {post}} \le \ell _1'$$ and
- $$\ell _2 \le \ell _j^{\text {cpre}}, \ell _j^{\text {cpost}} \le \ell _2'$$.

Intuitively, a conflict represents a *minimal violation* of a mutex constraint due to the execution of the statement at location $\ell _j^{\text {cpre}}$ in thread *j* between the two statements at locations $\ell _i^{\text {pre}}$ and $\ell _i^{\text {mid}}$ in thread *i*. Note that a statement at location $\ell$ in thread $tid$ is executed when the current location of $tid$ changes from $\ell$ to $\mathsf{succ}(\ell )$.

Given a conflict $$c = (\ell _i^{\text {pre}},\ell _i^{\text {mid}},\ell _i^{\text {post}},\ell _j^{\text {cpre}},\ell _j^{\text {cpost}})$$, let $$\text {pre}(c) = \ell _i^{\text {pre}}, \text {mid}(c) = \ell _i^{\text {mid}}, \text {post}(c) = \ell _i^{\text {post}}, \text {cpre}(c) = \ell _j^{\text {cpre}}$$ and $$\text {cpost}(c) = \ell _j^{\text {cpost}}$$. Further, let $$ tid _1(c) = i$$ and $$ tid _2(c) = j$$. To prune all interleavings prohibited by the mutex constraints from $${\mathsf {P}}'_{\textit{abs}}$$ we need to consider all conflicts derived from all mutex constraints. We denote this set as $$\mathbb {C}$$ and let $$K= |\mathbb {C}|$$.

##### Example

**Fig. 17:**
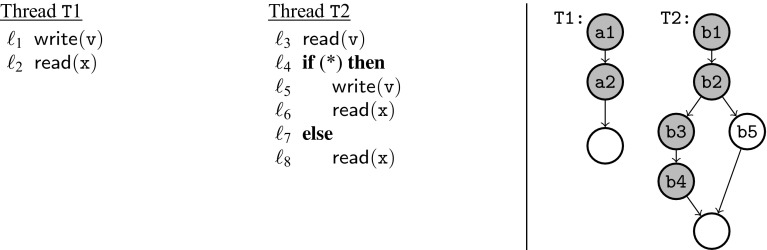
Example: mutex constraints and conflicts

We have an example program and its flow-graph in Fig. 17 (we skip the statement labels in the nodes here). Suppose in some iteration we obtain ${\mathrm {mtx}(\mathtt {T}1.[\ell _{1}{:}\ell _{2}],\mathtt {T}2.[\ell _{3}{:}\ell _{6}])}$. This yields 2 conflicts: $c_1$ given by $(\ell _{3},\ell _{4},\ell _{5},\ell _{1},\ell _{2})$ and $c_2$ given by $(\ell _{4},\ell _{5},\ell _{6},\ell _{1},\ell _{2})$. On an aside, this example also illustrates the difficulty of lock placement in the actual code. The mutex constraint would naïvely be translated to the lock ${\mathtt {lock}(\mathtt {T}1.[\ell _{1}:\ell _{2}],\mathtt {T}2.[\ell _{3}:\ell _{6}])}$. This is not a valid lock placement; in executions executing the else branch, the lock is never released.

#### Constructing new ${{\mathsf {P}}'_{\textit{abs}}}$

Initially, let NFA ${\mathsf {P}}'_{\textit{abs}}$ be given by the tuple $(Q_{\text {old}},{\varSigma }\cup \{\epsilon \},{\varDelta }_{\text {old}},Q_{\iota ,\text {old}},F_{\text {old}})$, where

- $Q_{\text {old}}$ is the set of states $\langle \mathscr {V}_o, \textit{ctid}, (\ell _1,\ldots ,\ell _n)\rangle$ of the abstract program $\mathscr {C}_{\textit{abs}}$ corresponding to $\mathscr {C}$, as well as $\langle \mathtt {terminated}\rangle$ and $\langle \mathtt {failed}\rangle$,
- ${\varSigma }$ is the set of abstract observable symbols,
- $Q_{\iota ,\text {old}}$ is the initial state of $\mathscr {C}_{\textit{abs}}$,
- $$F_{\text {old}}=\{\langle \mathtt {terminated}\rangle \}$$ and
- ${\varDelta }_{\text {old}} \subseteq Q_{\text {old}} \times {\varSigma }\cup \{\epsilon \} \times Q_{\text {old}}$ is the transition relation with $(q, \alpha , q') \in {\varDelta }_{\text {old}}$ iff $q \xrightarrow {\alpha } q'$ according to the abstract preemptive semantics.

To enable pruning paths that violate mutex constraints, we augment the state space of $${\mathsf {P}}'_{\textit{abs}}$$ to track the status of conflicts $$c_1,\ldots , c_K$$ using *four-valued* propositions $$p_1,\ldots , p_K$$, respectively. Initially all propositions are 0. Proposition $$p_k$$ is incremented from 0 to 1 when conflict $$c_k$$ is *activated*, i.e., when control moves from $$\ell _i^{\text {pre}}$$ to $$\ell _i^{\text {mid}}$$ along a path. Proposition $$p_k$$ is incremented from 1 to 2 when conflict $$c_k$$ *progresses*, i.e., when thread $$ tid _i$$ is at $$\ell _i^{\text {mid}}$$ and control moves from $$\ell _j^{\text {cpre}}$$ to $$\ell _j^{\text {cpost}}$$. Proposition $$p_k$$ is incremented from 2 to 3 when conflict $$c_k$$ *completes*, i.e., when control moves from $$\ell _i^{\text {mid}}$$ to $$\ell _i^{\text {post}}$$. In practice the value 3 is never reached because the state is pruned when the conflict completes. Proposition $$p_k$$ is reset to 0 when conflict $$c_k$$ is *aborted*, i.e., when thread $$ tid _i$$ is at $$\ell _i^{\text {mid}}$$ and either moves to a location different from $$\ell _i^{\text {post}}$$, or moves to $$\ell _i^{\text {post}}$$ before thread $$ tid _j$$ moves from $$\ell _j^{\text {cpre}}$$ to $$\ell _j^{\text {cpost}}$$.

##### Example

In Fig. 17, $c_1$ is activated when $\mathtt {T2}$ moves from $\mathtt {b1}$ to $\mathtt {b2}$; $c_1$ progresses if now $\mathtt {T1}$ moves from $\mathtt {a1}$ to $\mathtt {a2}$ and is aborted if instead $\mathtt {T2}$ moves from $\mathtt {b2}$ to $\mathtt {b3}$; $c_2$ completes after progressing if $\mathtt {T2}$ moves from $\mathtt {b2}$ to $\mathtt {b3}$ and is aborted if instead $\mathtt {T2}$ moves from $\mathtt {b2}$ to $\mathtt {b5}$.

Formally, the new ${\mathsf {P}}'_{\textit{abs}}$ is given by the tuple $(Q_{\text {new}},{\varSigma }\cup \{\epsilon \},{\varDelta }_{\text {new}},Q_{\iota ,new},F_{\text {new}})$, where:

- $$Q_{\text {new}} = Q_{\text {old}} \times \{0,1,2\}^K$$,
- ${\varSigma }$ is the set of abstract observable symbols as before,
- $$Q_{\iota ,\text {new}} = (Q_{\iota ,\text {old}},(0,\ldots ,0))$$,
- $$F_{\text {new}} = \{(Q, (p_1,\ldots ,p_K)): Q \in F_{\text {old}} \wedge p_1,\ldots ,p_K\in \{0,1,2\}\}$$ and
- ${\varDelta }_{\text {new}}$ is constructed as follows: add $((Q,(p_1,\ldots ,p_K)),\alpha , (Q',(p'_1,\ldots ,p'_K)))$ to ${\varDelta }_{\text {new}}$ iff $(Q, \alpha , Q') \in {\varDelta }_{\text {old}}$ and for each $k \in [1,K]$, the following hold:

1. *Conflict activation:* (the statement at location $$\text {pre}(c_k)$$ in thread $$ tid _1(c_k)$$ is executed) if $$p_k = 0, \textit{ctid} = \textit{ctid}' = tid _1(c_k), \ell _{\textit{ctid}} = \text {pre}(c_k)$$ and $$\ell '_{\textit{ctid}} = \text {mid}(c_k)$$, then $$p'_k = 1$$ else $$p'_k = 0$$,
2. *Conflict progress:* (thread $$ tid _1(c_k)$$ is interrupted by $$ tid _2(c_k)$$ and the conflicting statement at location $$\text {cpre}(c_k)$$ is executed) else if $$p_k = 1, \textit{ctid} = \textit{ctid}' = tid _2(c_k), \ell _{ tid _1(c_k)} = \ell '_{ tid _1(c_k)} = \text {mid}(c_k), \ell _{\textit{ctid}} = \text {cpre}(c_k)$$ and $$\ell '_{\textit{ctid}} = \text {cpost}(c_k)$$, then $$p'_k = 2$$,
3. *Conflict completion and state pruning:* (the statement at location $$\text {mid}(c_k)$$ in thread $$ tid _1(c_k)$$ is executed and that completes the conflict) else if $$p_k = 2, \textit{ctid} = \textit{ctid}' = tid _1(c_k), \ell _{\textit{ctid}} = \text {mid}(c_k)$$ and $$\ell '_{\textit{ctid}} = \text {post}(c_k)$$, then delete state $$(Q',(p'_1,\ldots ,p'_K))$$,
4. *Conflict abortion 1:* ($$ tid _1(c_k)$$ executes alternate statement) else if $$p_k = 1$$ or $$2, \textit{ctid} = \textit{ctid}' = tid _1(c_k), \ell _{\textit{ctid}} = \text {mid}(c_k)$$ and $$\ell '_{\textit{ctid}} \ne \text {post}(c_k)$$, then $$p'_k = 0$$,
5. *Conflict abortion 2:* ($$ tid _1(c_k)$$ executes statement at location $$\text {mid}(c_k)$$ without interruption by $$tid_2(c_k)$$) else if $$p_k = 1, \textit{ctid} = \textit{ctid}' = tid _1(c_k), \ell _{\textit{ctid}} = \text {mid}(c_k)$$ and $$\ell '_{\textit{ctid}} = \text {post}(c_k), \ell _{ tid _2(c_k)} = \ell '_{ tid _2(c_k)} = \text {cpre}(c_k)$$, then $$p'_k = 0$$

In our implementation, the new ${\mathsf {P}}'_{\textit{abs}}$ is constructed on-the-fly. Moreover, we do not maintain the entire set of propositions $p_1,\ldots ,p_K$ in each state of ${\mathsf {P}}'_{\textit{abs}}$. A proposition $p_i$ is added to the list of tracked propositions only after conflict $c_i$ is activated. Once conflict $c_i$ is aborted, $p_i$ is dropped from the list of tracked propositions.

##### Theorem 7

We are given a program $\mathscr {C}_{\textit{abs}}$ and a sequence of observable symbols $\omega$ that is a counterexample to preemption-safety, formally $\omega \in {\mathscr {L}}({\mathsf {P}}'_{\textit{abs}})\wedge \omega \notin \mathrm {Clo}_I( {\mathscr {L}}(\mathsf {NP}_{\textit{abs}}))$. If a pattern *P* eliminating $\omega$ is found, then, after enforcing all resulting mutex constraints in ${\mathsf {P}}'_{\textit{abs}}$, the counterexample $\omega$ is no longer accepted by ${\mathsf {P}}'_{\textit{abs}}$, formally $\omega \notin {\mathscr {L}}({\mathsf {P}}'_{\textit{abs}})$.

##### Proof

The pattern *P* eliminating $\omega$ represents a mutex constraint $\mathrm {mtx}( tid _i.[\ell _1{:}\ell ''_1], tid _j.[\ell _2{:}\ell ''_2])$, such that the trace $\omega$ is no longer possible. Mutex constraints represent conflicts of the form $(\ell _i^{\text {pre}},\ell _i^{\text {mid}},\ell _i^{\text {post}},\ell _j^{\text {cpre}},\ell _j^{\text {cpost}})$. Each such conflict represents a context switch that is not allowed: $\ell _i^{\text {pre}}\rightarrow \ell _i^{\text {mid}}\rightarrow \ell _j^{\text {cpre}}\rightarrow \ell _j^{\text {cpost}}\rightarrow \ell _i^{\text {mid}}\rightarrow \ell _i^{\text {post}}$. Because *P* eliminates $\omega$ we know that $\omega$ has a context switch from $tid _i.\ell '_1$ to $tid _j.\ell '_2$, where $\ell _1\le \ell '_1\le \ell ''_1$ and $\ell _2\le \ell '_2\le \ell ''_2$. One of the conflicts representing the mutex constraint is $(\ell _i^{\text {pre}},\ell _i^{\text {mid}},\ell _i^{\text {post}},\ell _j^{\text {cpre}},\ell _j^{\text {cpost}})$, where $\ell _i^{\text {mid}}=\ell '_1$ and $\ell _i^{\text {pre}}$ and $\ell _i^{\text {post}}$ are the locations immediately before and after $\ell '_1$. Further, $\ell _j^{\text {cpre}}=\ell '_2$ and $\ell _j^{\text {cpost}}$ the location immediately following $\ell '_2$. If now a context switch happens at location $\ell '_1$ switching to location $\ell '_2$, this triggers the conflict and this trace will be discarded in ${\mathsf {P}}'_{\textit{abs}}$. $\square$

## Global lock placement constraints

Our synthesis loop will keep collecting and enforcing conflicts ${\mathsf {P}}'_{\textit{abs}}$ until the language inclusion check holds. At that point we have collected a set of conflicts $\mathbb {C}_{\text {all}}$ that need to be enforced in the original program source code. To avoid deadlocks, the lock placement has to conform to a number of constraints.

We encode the global lock placement constraints for ensuring correctness as an SMT^3^ formula $\mathsf {LkCons}$. Let ${\mathsf {L}}$ denote the set of all location and $\mathsf {Lk}$ denote the set of all locks available for synthesis. We use scalars $\ell , \ell ', \ell _1, \ldots$ of type ${\mathsf {L}}$ to denote locations and scalars $LkVar , LkVar ', LkVar _1, \ldots$ of type $\mathsf {Lk}$ to denote locks. The number of locks is finite and there is a fixed locking order. Let $\mathsf {Pre}(\ell )$ denote the set of all immediate predecessors in node $\ell : \mathsf {stmt}({\ell })$ in the flow-graph of the original concrete concurrent program $\mathscr {C}$. We use the following Boolean variables in the encoding.

**Table:** 

$\mathsf {LockBefore}(\ell , LkVar )$	$\mathsf {lock}( LkVar )$ is placed just before the statement represented by $\ell$
$\mathsf {LockAfter}(\ell , LkVar )$	$\mathsf {lock}( LkVar )$ is placed just after the statement represented by $\ell$
$\mathsf {UnlockBefore}(\ell , LkVar )$	$\mathsf {unlock}( LkVar )$ is placed just before the statement represented by $\ell$
$\mathsf {UnlockAfter}(\ell , LkVar )$	$\mathsf {unlock}( LkVar )$ is placed just after the statement represented by $\ell$

For every location $\ell$ in the source code we allow a lock to be placed either immediately before or after it. If a lock $LkVar$ is placed before $\ell$, than $\ell$ is protected by $LkVar$. If $LkVar$ is placed after $\ell$, than $\ell$ is not protected by $LkVar$, but the successor instructions are. Both options are needed, e.g. to lock before the first statement of a thread and to unlock after the last statement of a thread. We define three additional Boolean variables:

1. $\mathsf {InLock}(\ell , LkVar )$: If location $\ell$ has no predecessor than it is protected by $LkVar$ if there is a lock statement before $\ell$.
  $$\begin{aligned} \mathsf {InLock}(\ell , LkVar ) = \mathsf {LockBefore}(\ell , LkVar ) \end{aligned}$$
  If there exists a predecessor $\ell '$ to $\ell$ than $\ell$ is protected by $LkVar$ if either there is a lock statement before $\ell$ or if $\ell '$ is protected by $LkVar$ and there is no unlock in between.
  $$\begin{aligned}&\mathsf {InLock}(\ell , LkVar ) = \mathsf {LockBefore}(\ell , LkVar ) \\&\quad \vee \ (\lnot \mathsf {UnlockBefore}(\ell , LkVar )\wedge \mathsf {InLockEnd}(\ell ', LkVar )) \end{aligned}$$
  Note that either all predecessors are protected by a lock or none. We enforce this in Rule C (*C7*) below.
2. $\mathsf {InLockEnd}(\ell , LkVar )$: The successors of $\ell$ are protected by $LkVar$ if either location $\ell$ is protected by $LkVar$ or $\mathsf {lock}( LkVar )$ is placed after $\ell$.
  $$\begin{aligned} (\mathsf {InLock}(\ell , LkVar ) \wedge \lnot \mathsf {UnlockAfter}(\ell , LkVar )) \vee \mathsf {LockAfter}(\ell , LkVar ) \end{aligned}$$
3. $$\mathsf {Order}( LkVar , LkVar ')$$: We give a fixed lock order that is transitive, asymmetric, and irreflexive. $$\mathsf {Order}( LkVar , LkVar ')=\mathtt {true}$$ iff $$ LkVar $$ needs to be acquired before $$ LkVar '$$. This means that an instruction $$\mathsf {lock}( LkVar )$$ cannot be place inside the scope of $$ LkVar '$$.

We describe the constraints and their SMT formulation constituting $\mathsf {LkCons}$ below. All constraints are quantified over all $\ell , \ell ', \ell _1, \ldots \in {\mathsf {L}}$ and all $LkVar , LkVar ', LkVar _1, \ldots \in \mathsf {Lk}$.

1. All locations in the same conflict in $\mathbb {C}_{\text {all}}$ are protected by the same lock.
  $$\begin{aligned} \forall \mathbb {C}\in \mathbb {C}_{\text {all}}:\ \ell ,\ell '\in \mathbb {C}\Rightarrow \exists LkVar .\ \mathsf {InLock}(\ell , LkVar )\wedge \mathsf {InLock}(\ell ', LkVar ) \end{aligned}$$
2. Placing $\mathsf {lock}( LkVar )$ immediately before/after $\mathsf {unlock}( LkVar )$ is disallowed. Doing so would make (*C1*) unsound, as two adjacent locations could be protected by the same lock and there could still be a context-switch in between because of the immediate unlocking and locking again. If $\ell$ has a predecessor $\ell '$ then
  $$\begin{aligned} \mathsf {UnlockBefore}(\ell , LkVar ) \Rightarrow&(\lnot \mathsf {LockAfter}(\ell ', LkVar ))\\ \mathsf {LockBefore}(\ell , LkVar ) \Rightarrow&(\lnot \mathsf {UnlockAfter}(\ell ', LkVar )) \end{aligned}$$
3. We enforce the lock order according to $\mathsf {Order}$ defined in (*D3*).
  $$\begin{aligned} \mathsf {LockAfter}(\ell , LkVar ) \wedge \mathsf {InLock}(\ell , LkVar ') \Rightarrow \mathsf {Order}( LkVar ', LkVar )\\ \mathsf {LockBefore}(\ell , LkVar ) \wedge \left( \underset{\ell ' \in \mathsf {Pre}(x)}{\bigvee }\mathsf {InLockEnd}(\ell ', LkVar ')\right) \Rightarrow \mathsf {Order}( LkVar ', LkVar ) \end{aligned}$$
4. Existing locks may not be nested inside synthesized locks. They are implicitly ordered before synthesized locks in our lock order.
  $$\begin{aligned} (\mathsf {stmt}({\ell }) = \mathsf {lock}(\ldots )) \Rightarrow \lnot \mathsf {InLock}(\ell , LkVar ) \end{aligned}$$
5. No $\mathsf {wait}$ statements may be in the scope of synthesized locks to prevent deadlocks.
  $$\begin{aligned} (\mathsf {stmt}({\ell }) = \mathsf {wait}(\ldots )/\mathsf {wait\_not}(\ldots )/\mathsf {wait\_reset}(\ldots )) \Rightarrow \lnot \mathsf {InLock}(\ell , LkVar ) \end{aligned}$$
6. Placing both $\mathsf {lock}( LkVar )$ and $\mathsf {unlock}( LkVar )$ before/after $\ell$ is disallowed.
  $$\begin{aligned}&(\lnot \mathsf {LockBefore}(\ell , LkVar ) \vee \lnot \mathsf {UnlockBefore}(\ell , LkVar ))\; \wedge \\&(\lnot \mathsf {LockAfter}(\ell , LkVar ) \vee \lnot \mathsf {UnlockAfter}(\ell , LkVar )) \end{aligned}$$
7. All predecessors must agree on their $\mathsf {InLockEnd}$ status. This ensures that joining branches hold the same set of locks. If $\ell$ has at least one predecessor then
  $$\begin{aligned} \left( \underset{\ell ' \in \mathsf {Pre}(x)}{\bigwedge }\ \mathsf {InLockEnd}(\ell ', LkVar )\right) \; \vee \left( \underset{\ell ' \in \mathsf {Pre}(x)}{\bigwedge }\ \lnot \mathsf {InLockEnd}(\ell ', LkVar )\right) \end{aligned}$$
8. $\mathsf {unlock}( LkVar )$ can only be placed only after a $\mathsf {lock}( LkVar )$.
  $$\begin{aligned} \mathsf {UnlockAfter}(\ell , LkVar ) \Rightarrow \mathsf {InLock}(\ell , LkVar ) \end{aligned}$$
  If $\ell$ has a predecessor $\ell '$ then also
  $$\begin{aligned} \mathsf {UnlockBefore}(\ell , LkVar ) \Rightarrow \mathsf {InLockEnd}(\ell ', LkVar ) \end{aligned}$$
  else if $\ell$ has no predecessor then
  $$\begin{aligned} \mathsf {UnlockBefore}(\ell , LkVar ) = \mathsf {false}\end{aligned}$$
9. We forbid double locking: A lock may not be acquired if that location is already protected by the lock.
  $$\begin{aligned} \mathsf {LockAfter}(\ell , LkVar ) \Rightarrow \lnot \mathsf {InLock}(\ell , LkVar ) \end{aligned}$$
  If $\ell$ has a predecessor $\ell '$ then also
  $$\begin{aligned} LockBefore(\ell , LkVar ) \Rightarrow \lnot \mathsf {InLockEnd}(\ell , LkVar ) \end{aligned}$$
10. The end state $\mathsf{last}_i$ of thread *i* is unlocked. This prevents locks from leaking.
  $$\begin{aligned} \forall i:\lnot \mathsf {InLock}(\mathsf{last}_i,lk) \end{aligned}$$

According to constraints (*C4*) and (*C5*) no locks may be placed around existing $\mathsf {wait}$ or $\mathsf {lock}$ statements. Since both statements are implicit preemption points, where the non-preemptive semantics may context-switch, it is never necessary to synthesize a lock across an existing $\mathsf {lock}$ or $\mathsf {wait}$ instruction to ensure preemption-safety.

We have the following result.

### Theorem 8

Let concurrent program $\mathscr {C}'$ be obtained by inserting any lock placement satisfying $\mathsf {LkCons}$ into concurrent program $\mathscr {C}$. Then $\mathscr {C}'$ is guaranteed to be preemption-safe w.r.t. $\mathscr {C}$ and not to introduce new deadlocks (that were not already present in $\mathscr {C}$).

### Proof

To show preemption-safety we need to show that language inclusion holds (Proposition 1). Language inclusion follows directly from constraint (*C1*), which ensures that all mutex constraints are enforced as locks. Further, constraints (*C2*) and (*C6*) ensure that there is never a releasing and immediate reacquiring of locks in between statements. This is crucial because otherwise a context-switch in between two instructions protected by a lock would be possible.

Let as assume towards contradiction that a new deadlocked state $s=\langle \mathscr {V}, \textit{ctid}, (\ell _1,\ldots ,\ell _n)\rangle$ is reachable in $\mathscr {C}'$. By definition this means that none of the rules of the preemptive semantics of ${\mathscr {W}}$ (Figs. 7, 8) are applicable in *s*. Remember, that an infinite loop is considered a lifelock. We proceed to enumerate all rules of the preemptive semantics that may block:

- All threads reached their $\mathsf{last}$ location, then the Terminate rule is the only one that could be applicable. If it is not, then a lock is still locked. This deadlock is prevented by condition (*C10*).
- The rule Nswitch is not applicable because the other thread is blocked and Seq is not applicable because none of the rules of the single-thread semantics (Fig. 6) apply. The following sequential rules have preconditions that may prevent them from being applicable.
- Rule Lock may not proceed if the lock $$ LkVar $$ is taken. If $$ LkVar ={\textit{ctid}}$$ we have a case of double-locking that is prevented by constraint (*C9*). Otherwise $$ LkVar =j\ne \textit{ctid}$$. In this case $$ tid _{\textit{ctid}}$$ is waiting for $$ tid _j$$. This may be because of
  - a circular dependency of locks. This cannot be a new deadlock because of constraints (*C4*) and (*C3*) enforcing a strict lock order even w.r.t. existing locks.
  - another deadlock in $$ tid _j$$. This deadlock cannot be new because we can make a recursive argument about the deadlock in $$ tid _j$$.
- Rule Unlock may not proceed if the lock is not owned by the executing thread. In this case we either have a case of double-unlock (prevented by constraint (*C8*)) or a lock is unlocked that is not held by $tid _{\textit{ctid}}$ at that point. The latter may happen because the lock was not taken on all control flow paths leading to $\ell _{\textit{ctid}}$. This is prevented by constraints (*C7*) and (*C8*).
- Rules Wait/Wait_not/Wait_reset may not proceed if the condition variable is not in the right state. According to constraint (*C5*) $\ell _{\textit{ctid}}$ cannot be protected by a synthesized lock. This means the deadlock is either not new or it is caused by a deadlock in a different thread making it impossible to reach $\mathsf {signal}( CondVar )/\mathsf {reset}( CondVar )$. In that case a recursive argument applies.
- The Thread_end rule is not applicable because all other threads are blocked. This is impossible by the same reasoning as above. $\square$

## Optimizing lock placement

The global lock placement constraint $\mathsf {LkCons}$ constructed in Sect. 8 often has multiple models corresponding to very different lock placements. The desirability of these lock placements varies considerably due to performance considerations. For example a coarse-grained lock placement may be useful when the cost of locking operations is relatively high compared to the cost of executing the critical sections, while a fine-grained lock placement should be used when locking operations are cheap compared to the cost of executing the critical sections. Neither of these lock placement strategies is guaranteed to find the optimally performing program in all scenarios. It is necessary for the programmer to judge when each criterion is to be used.

Here, we present objective functions *f* to distinguish between different lock placements. Our synthesis procedure combines the function *f* with the global lock placement constraints $\mathsf {LkCons}$ into a single maximum satisfiability modulo theories (MaxSMT) problem and the optimal model corresponds to the *f*-optimal lock placement. We present objective functions for coarse- and fine-grained locking.

### Objective functions

We say that a statement $\ell : stmt$ in a concurrent program $\mathscr {C}$ is protected by a lock $LkVar$ if $\mathsf {InLock}(\ell , LkVar )$ is true. We define the two objective functions as follows:

1. *Coarsest-grained locking* This objective function prefers a program $\mathscr {C}_1$ over $\mathscr {C}_2$ if the number of lock statements in $\mathscr {C}_1$ is fewer than in $\mathscr {C}_2$. Among the programs having the same number of lock statements, the ones with the fewest statements protected by any lock are preferred. Formally, we can define $\mathsf {Coarse}(\mathscr {C}_i)$ to be $\lambda + \epsilon \cdot \mathsf {StmtInLock}(\mathscr {C}_i)$ where $\lambda$ is the count of lock statements in $\mathscr {C}_i, \mathsf {StmtInLock}(\mathscr {C}_i)$ is the count of statements in $\mathscr {C}_i$ that are protected by any lock and $\epsilon$ is given by $\frac{1}{2k}$ where *k* is the total number of statements in $\mathscr {C}_i$. The reasoning behind this formula is that the total cost is always dominated by the number of lock statements. So if all statements are protected by a lock this fact contributes $\frac{1}{2}$ to the total cost.
2. *Finest-grained locking* This objective function prefers a program $\mathscr {C}_1$ over $\mathscr {C}_2$ if $\mathscr {C}_1$ allows more concurrency than $\mathscr {C}_2$. Concurrency of a program is measured by the number of pairs of statements from different threads that cannot be executed together. Formally, we define $\mathsf {Fine}(\mathscr {C}_i)$ to be the total number of pairs of statements ${\ell _1: stmt _1}$ and ${\ell _2: stmt _2}$ from different threads that cannot be executed at the same time, i.e., are protected by the same lock.

### Optimization procedure

The main idea behind the optimization procedure for the above objective functions is to build an instance of the MaxSMT problem using the global lock placement constraint $\mathsf {LkCons}$ such that (a) every model of $\mathsf {LkCons}$ is a model for the MaxSMT problem and the other way round; and (b) the cost of each model for the MaxSMT problem is the cost of the corresponding locking scheme according to the chosen objective function. The optimal lock placement is then computed by solving the MaxSMT problem.

A MaxSMT problem instance is given by $\langle {\varPhi }, \langle ({\varPsi }_1, w_1), \ldots \rangle \rangle$ where ${\varPhi }$ and each ${\varPsi }_i$ are SMT formulas and each $w_i$ is a real number. The formula ${\varPhi }$ is called the *hard constraint*, and each ${\varPsi }_i$ is called a *soft constraint* with *associated weight* $w_i$. Given an assignment *V* of variables occurring in the constraints, its cost *c* is defined as the sum of the weights of soft constraints that are falsified by $V: c = \sum _{i : V \not \models {\varPsi }_i} w_i$. The objective of the MaxSMT problem is to find a model that satisfies ${\varPhi }$ with minimal cost. Intuitively, by minimizing the cost we maximize the sum of the weights of the satisfied soft constraints.

In the following, we write $\mathsf {InLock}(\ell )$ as a short-hand for $\bigvee _{ LkVar } \mathsf {InLock}(\ell , LkVar )$, and similarly $\mathsf {LockBefore}(\ell )$ and $\mathsf {LockAfter}(\ell )$. For each of our two objective functions, the hard constraint for the MaxSMT problem is $\mathsf {LkCons}$ and the soft constraints and associated weights are as specified below:

- For the coarsest-grained locking objective function, the soft constraints are of three types: (a) $\lnot \mathsf {LockBefore}(\ell )$ with weight 1, (b) $\lnot \mathsf {LockAfter}(\ell )$ with weight 1, and (c) $\lnot \mathsf {InLock}(\ell )$ with weight $\epsilon$, where $\epsilon$ is as defined above.
- For the finest-grained locking objective function, the soft constraints are given by $\bigwedge _{lk} \lnot \mathsf {InLock}(\ell , lk) \vee \lnot \mathsf {InLock}(\ell ', lk)$, for each pair of statements $\ell$ and $\ell '$ from different threads. The weight of each soft constraint is 1.

#### Theorem 9

For the coarsest-grained and finest-grained objective functions, the cost of the optimal program is equal to the cost of the model for the corresponding MaxSMT problem obtained as described above.

## Implementation and evaluation

In order to evaluate our synthesis procedure, we implemented it in a tool called Liss, comprised of 5400 lines of C++ code. Liss uses Clang/LLVM 3.6 to parse C code and insert locks into the code. By using Clang’s rewriter, Liss is able to maintain the original formatting of the source code. As a MaxSMT solver, we use Z3 version 4.4.1 (unstable branch). Liss is available as open-source software along with benchmarks.^4^ The language inclusion algorithm is available separately as a library called Limi.^5^ Liss implements the synthesis method presented in this paper with several optimizations. For example, we take advantage of the fact that language inclusion violations can often be detected by exploring only a small fraction of $\mathsf {NP}_{\textit{abs}}$ and ${\mathsf {P}}'_{\textit{abs}}$, which we construct on the fly.

Our prototype implementation has some limitations. First, Liss uses function inlining during the analysis phase and therefore cannot handle recursive programs. During lock placement, however, functions are taken into consideration and it is ensured that a function does not “leak” locks. Second, we do not implement any form of alias analysis, which can lead to unsound abstractions. For example, we abstract statements of the form “*x = 0” as writes to variable x, while in reality other variables can be affected due to pointer aliasing. We sidestep this issue by manually massaging input programs to eliminate aliasing. This is not a limitation of our technique, which could be combined with known aliasing analysis techniques.

We evaluate our synthesis method w.r.t. the following criteria: (1) Effectiveness of synthesis from implicit specifications; (2) Efficiency of the proposed synthesis procedure; (3) Effectiveness of the proposed coarse abstraction scheme; (4) Quality of the locks placed.

### Benchmarks

We ran Liss on a number of benchmarks, summarized in Table 1. For each benchmark we report the complexity [lines of code (LOC), number of threads (Th)], the number of iterations (It) of the language inclusion check (Fig. 12) and the maximum bound *k* (MB) that was used in any iteration of the language inclusion check. Further we report the total time (TT) taken by the language inclusion check loop and the time for solving the MaxSMT problem for the two objective functions (Coarse, Fine). Finally, we report the maximum resident set size (Memory). All measurements were done on an Intel core i5-3320M laptop with 8 GB of RAM under Linux.

**Table 1:** Experiments

Name	LOC	Th	It	MB	TT (s)	Coarse (s)	Fine (s)	Memory (MB)	CR (s)
ConRepair benchmarks									
ex1.c	18	2	1	1	<1	<1	<1	29	<1
ex2.c	23	2	1	1	<1	<1	<1	29	<1
ex3.c	37	2	1	1	<1	<1	<1	29	<1
ex5.c	42	2	4	1	<1	<1	<1	32	<1
$\hbox {lc-rc.c}^\mathrm{c}$	35	4	0	1	<1	N/A	N/A	15	9
dv1394.c	37	2	2	1	<1	<1	<1	32	17
em28xx.c	20	2	1	1	<1	<1	<1	29	<1
f_acm.c	54	3	6	1	<1	<1	<1	35	1872
i915_irq.c	17	2	1	1	<1	<1	<1	29	2.6
ipath.c	23	2	1	3	<1	<1	<1	29	12
iwl3945.c	26	3	0	1	<1	<1	<1	15	5
md.c	35	2	1	1	<1	<1	<1	30	1.5
$\hbox {myri10ge.c}^\mathrm{c}$	60	4	0	3	<1	N/A	N/A	16	1.5
usb-serial.bug1.c	357	7	2	1	6.1	<1	<1	267	$\infty ^\mathrm{b}$
usb-serial.bug2.c	355	7	2	1	4.5	<1	<1	175	3563
usb-serial.bug3.c	352	7	2	1	2.8	<1	<1	105	$\infty ^\mathrm{b}$
usb-serial.bug4.c	351	7	2	1	3.8	<1	<1	130	$\infty ^\mathrm{b}$
$\hbox {usb-serial.c}^\mathrm{a}$	357	7	0	3	31.9	N/A	N/A	792	1200
CPMAC driver benchmark									
cpmac.bug1.c	1275	5	1	2	6	1.6	1.1	156	
cpmac.bug2.c	1275	5	4	10	152.9	63	41.4	1210	
cpmac.bug3.c	1270	5	9	4	11.1	16.2	9.6	521	
cpmac.bug4.c	1276	5	4	7	107.3	10.5	6.5	5392	
cpmac.bug5.c	1275	5	4	4	136.5	11	7.7	3549	
$\hbox {cpmac.c}^\mathrm{a}$	1276	5	0	1	2.1	N/A	N/A	114	
memcached benchmark									
memcached.c	294	2	104	2	22.8	6.2	2.1	114	

*Th* threads, *It* iterations, *MB* max bound, *TT* time for language incl. loop, *CR* ConRepair time

$^\mathrm{a}$ Bug-free example

$$^\mathrm{b}$$ Timeout after 3 h

$^\mathrm{c}$ Race not detected, as it was present under non-preemptive scheduling

**Table 2:** Lock placement statistics: the number of synthesized lock variables, lock and unlock statements, and the number of abstract statements protected by locks for different objective functions

Name	No objective			$\mathsf {Coarse}$			$\mathsf {Fine}$		
	Locks	locks/unlocks	Protected instr	Locks	locks/unlocks	Protected instr	Locks	locks/unlocks	Protected instr
cpmac.bug1	2	6/6	11	1	3/3	11	1	3/3	9
cpmac.bug2	2	22/23	119	1	4/4	98	1	6/7	95
cpmac.bug3	1	4/4	29	1	2/3	29	1	5/6	28
cpmac.bug4	4	16/16	53	1	4/4	53	1	6/6	26
cpmac.bug5	3	15/15	30	1	4/4	30	1	5/5	30
memcached	2	5/5	26	1	1/1	28	1	2/2	24

#### Implicit versus explicit specification

In order to evaluate the effectiveness of synthesis from implicit specifications, we apply Liss to the set of benchmarks used in our previous ConRepair tool for assertion-based synthesis [6]. In addition, we evaluate Liss and ConRepair on several *new* assertion-based benchmarks (Table 1). We report the time ConRepair took in Table 2. We added $\mathsf {yield}$ statements to the source code of the benchmarks to indicate where a context-switch in the driver would be expected by the developer. This is a very light-weight annotation burden compared to the assertions required by ConRepair.

The set includes synthetic microbenchmarks modeling typical concurrency bug patterns in Linux drivers and the usb-serial macrobenchmark, which models a complete synchronization skeleton of the USB-to-serial adapter driver. For Liss we preprocess these benchmarks by eliminating assertions used as explicit specifications for synthesis. In addition, we replace statements of the form assume(v) with await(v), redeclaring all variables v used in such statements as condition variables. This is necessary as our program syntax does not include assume statements.

We use Liss to synthesize a preemption-safe, deadlock-free version of each benchmark. This method is based on the assumption that the benchmark is correct under non-preemptive scheduling and bugs can only arise due to preemptive scheduling. We discovered two benchmarks (lc-rc.c and myri10ge.c) that violated this assumption, i.e., they contained bugs that manifested under non-preemptive scheduling; Liss did not detect these bugs. Liss was able to detect and fix all other known races without relying on assertions. Furthermore, Liss detected a new race in the usb-serial family of benchmarks, which was not detected by ConRepair due to a missing assertion.

##### 10.1.1.1 Performance and precision

ConRepair uses CBMC for verification and counterexample generation. Due to the coarse abstraction we use, both are much cheaper with Liss. For example, verification of usb-serial.c, which was the most complex in our set of benchmarks, took Liss 103 s, whereas it took ConRepair 20 min [6].

The MaxSMT lock placement problem is solved in less than 65s for our choice of objective functions. It is clear that without an objective function the lock placement problem is in SAT, and Z3 solves it in less than 1s in each case. The coarse- and fine-grained lock placement are natural choices, we did not attempt other more involved objective functions.

The loss of precision due to abstraction may cause the inclusion check to return a counterexample that is spurious in the concrete program, leading to unnecessary synchronization being synthesized. On our existing benchmarks, this only occurred once in the usb-serial driver, where abstracting away the return value of a function led to an infeasible trace. We refined the abstraction manually by introducing a guard variable to model the return value.

#### Simplified real-world benchmarks

In this section we present two additional benchmarks derived from real-world concurrent programs. Both benchmarks were manually preprocessed to eliminate pointer aliasing.

##### 10.1.2.1 CPMAC benchmark

This benchmark is based on a complete Linux driver for the TI AR7 CPMAC Ethernet controller. The benchmark was constructed as follows. We combined the driver with a model of the OS API and the software interface of the device written in C. We modeled most OS API functions as writes to a special memory location. Groups of unrelated functions were modeled using separate locations. Slightly more complex models were required for API functions that affect thread synchronization. For example, the free_irq function, which disables the driver’s interrupt handler, blocks, waiting for any outstanding interrupts to finish. Drivers can rely on this behavior to avoid races. We introduced a condition variable to model this synchronization. Similarly, most device accesses were modeled as writes to a special ioval variable. Thus, the only part of the device that required a more accurate model was its interrupt enabling logic, which affects the behavior of the driver’s interrupt handler thread.

Our original model consisted of eight threads. Liss ran out of memory on this model, so we simplified it to five threads by eliminating parts of driver functionality. Nevertheless, we believe that the resulting model represents the most complex synchronization synthesis case study, based on real-world code, reported in the literature.

The CPMAC driver used in this case study did not contain any known concurrency bugs, so we artificially simulated five typical concurrency bugs that commonly occur in drivers of this type [5]: a data race where two threads could be concurrently modifying the hardware packet queue, leaving it in invalid state; an IRQ race where driver resources were deallocated while its interrupt handler could still be executing, leading to a use-after-free error; an initialization race where the driver’s request queue was enabled before the device was fully initialized, and two races between interrupt enable and disable operations, causing the driver to freeze. Liss was able to detect and automatically fix each of these defects (bottom part of Table 1). We only encountered two program locations where manual abstraction refinement was necessary. These results support our choice of data-oblivious abstraction and confirm the conjecture that synchronization patterns in OS code rarely depend on data values. At the same time, the need for manual refinements indicates that achieving fully automatic synthesis requires enhancing our method with automatic abstraction refinement.

##### 10.1.2.2 Memcached benchmark

Finally, we evaluate Liss on *memcached*, an in-memory key-value store version 1.4.5 [19]. The core of memcached is a non-reentrant library of store manipulation primitives. This library is wrapped into the thread.c module that implements a thread-safe API used by server threads. Each API function performs a sequence of library calls protected with locks. In this case study, we synthesize lock placement for a fragment of the thread.c module. In contrast to our other case studies, here we would like to synthesize locking from scratch rather than fix defects in existing lock placement. Furthermore, optimal locking strategy in this benchmark depends on the specific load. We envision that the programmer may synthesize both a coarse-grained and a fine-grained version and at deployment the appropriate version is selected.

#### Quality of synthesis

Next, we focus on the quality of synthesized solutions for the two real-world benchmarks from our benchmark set. Table 2 compares the implementation synthesized for these benchmarks using each objective functions in terms of (1) the number of locks used in synthesized code, (2) the number of lock and unlock statements generated, and (3) total number of program statements protected by synthesized locks.

We observe that different objective functions produce significantly different results in terms of the size of synthesized critical sections and the number of lock and unlock operations guarding them: the fine-grained objective synthesizes smaller critical sections at the cost of introducing a larger number of lock and unlock operations. Implementations synthesized without an objective function are clearly of lower quality than the optimized versions: they contains large critical sections, protected by unnecessarily many locks. These observations hold for the CPMAC benchmarks, where we start with a program that has most synchronization already in place, as well as the memcached benchmark, where we synthesize synchronization from scratch.

To summarize our experiments, we found that (1) while our coarse abstraction is highly precise in practice, automatic abstraction refinement is required to further reduce manual effort involved in synchronization synthesis; we leave such extension to future work; (2) additional work is required to improve the performance of our method to be able to handle real-world systems without simplification; (3) the objective functions allow specializing synthesis to a particular locking scheme; (4) the time required to solve the MaxSMT problem is small compared to the analysis time.

## Conclusion

We introduced a technique to synthesize locks using an implicit specification. The implicit specification relieves the programmer of the burden of providing sufficient assertions to specify correctness of the program. Our synthesis is guaranteed not to introduce deadlocks and the lock placement can be optimized using a static optimization function.

In ongoing work [7] we aim to optimize lock placement not merely using syntactic criteria, but by optimizing the actual performance of the program running on a specific system. In this approach we start with a synthesized program that uses coarse locking and then profile the performance on a real system. Using those measurements we adjust the locking to be more fine-grained in those areas where a high contention was measured.

## Footnotes

1 An expression/assignment statement that involves reading from/writing to multiple shared variables can always be rewritten into a sequence of atomic read/atomic write statements using local variables. For example the statement $\mathtt {x}:=\mathtt {x}+1$, where x is a global variable can be translated to $\mathtt {l}=\mathtt {x};\ \mathtt {x}=\mathtt {l}+1$, where l is a fresh local variable.

2 The equivalence classes of $\approx ^t_I$ are Mazurkiewicz traces.

3 The encoding of the global lock placement constraints is essentially a SAT formula. We present and use this as an SMT formula to enable combining the encoding with objective functions for optimization (see Sect. 9).

4
https://github.com/thorstent/Liss.

5
https://github.com/thorstent/Limi.
